# Tirzepatide Attenuates Pulmonary Vascular Remodeling in Pulmonary Arterial Hypertension: Association with the SIRT1/H4K12la/c-Jun Axis

**DOI:** 10.3390/cells15201900

**Published:** 2026-10-19

**Authors:** Sutong Zhan, Yong Deng, Peilin Chen, Tangfeng Lv, Yong Song, Jiaxin Liu, Chuiyu Kong

**Affiliations:** 1 Department of Respiratory and Critical Care Medicine, Affiliated Jinling Hospital, Medical School of Nanjing University, Nanjing 210000, China; 2 Department of Cardiac Surgery, Nanjing Drum Tower Hospital, Affiliated Hospital of Medical School, Nanjing University, Nanjing 210000, Chinacykong@njmu.edu.cn (C.K.); 3 Department of Respiratory and Critical Care Medicine, Nanjing Drum Tower Hospital, Affiliated Hospital of Medical School, Nanjing University, Nanjing 210000, China

**Keywords:** pulmonary arterial hypertension, tirzepatide, histone lactylation, phenotypic switching, metabolic reprogramming

## Abstract

Metabolic reprogramming and the phenotypic switching of pulmonary arterial smooth muscle cells (PASMCs) are critical pathological features of pulmonary arterial hypertension (PAH). Here, we demonstrated that Tirzepatide (TZP) can significantly ameliorate echocardiographic indicators of pulmonary vascular load and attenuate distal pulmonary vascular remodeling by suppressing PASMC phenotypic switching. Metabolomics and cellular metabolic flux analyses revealed that TZP corrected the Warburg effect, thereby attenuating intracellular lactate accumulation, which we identified as a driver that promotes the synthetic phenotype in PASMCs and exacerbates PAH. Mechanistically, the protective effects of TZP were strongly associated with the activation of the AMPK/SIRT1 signaling pathway, which mediated a significant reduction in histone H4 lysine 12 lactylation (H4K12la). This epigenetic reduction suppressed the transcription of the proto-oncogene *Jun* and its downstream pathways, which promote PASMC phenotypic switching and PAH deterioration. Thus, we propose a working model wherein TZP attenuates PAH progression by modulating pathological metabolism and reducing c-Jun transcription via the AMPK/SIRT1/H4K12la axis. These findings provide preliminary translational implications for targeting metabolic-epigenetic crosstalk and offer a preclinical rationale for exploring TZP as a protective pharmacological strategy in PAH.

## 1. Introduction

Pulmonary arterial hypertension (PAH) is a fatal cardiovascular disease characterized by a progressive increase in pulmonary vascular resistance, which frequently culminates in right heart failure and death [1]. Pulmonary vascular remodeling is the core pathological hallmark of PAH, primarily driven by the abnormal proliferation, migration, and phenotypic transition of pulmonary artery smooth muscle cells (PASMCs) [2]. During the progression of PAH, PASMCs undergo an irreversible phenotypic switch from a quiescent contractile state to a hyperproliferative synthetic state, accompanied by excessive extracellular matrix (ECM) deposition [3,4]. Therefore, elucidating the key molecular mechanisms capable of reversing the PASMC phenotypic switch is of paramount importance for the development of novel targeted therapeutic strategies for PAH.

In recent years, the role of metabolic reprogramming in regulating the PASMC phenotypic switch has garnered widespread attention. Similar to the Warburg effect observed in cancer cells, the energy metabolism of PAH-PASMCs shifts predominantly from mitochondrial oxidative phosphorylation to aerobic glycolysis, even under normoxic conditions [5]. This metabolic shift not only drives glycolysis-induced medial hypertrophy but also confers apoptosis resistance to the pulmonary vasculature, fundamentally contributing to vascular remodeling in PAH [6]. In addition, this relentless glycolytic flux inevitably results in the massive accumulation of downstream metabolites, particularly lactate. While traditionally considered merely a metabolic byproduct of glycolysis, emerging evidence indicates that lactate can act as a crucial epigenetic regulatory molecule, directly modulating gene transcription through histone lactylation [7]. Previous studies have established that histone lactylation acts as a crucial epigenetic driver for the phenotypic switching of vascular smooth muscle cells (VSMCs), playing an indispensable role in the development of vascular disorders like atherosclerosis and aortic dissection [8,9]. In pulmonary hypertension, a recent study by Yang et al. demonstrated that lactate accumulation-induced histone lactylation at the PDE4B promoter drives the transcriptional expression of downstream target genes, leading to PASMC proliferation and migration [10]. However, whether histone lactylation participates in the phenotypic switch of PAH-PASMCs, as well as the specific downstream transcriptional regulatory networks involved, remain largely unknown.

Plenty of studies have highlighted that metabolic syndromes, such as obesity, obstructive sleep apnea (OSA), and insulin resistance, are significant risk factors contributing to the pathogenesis and progression of PAH [11,12,13]. Accordingly, pharmacological correction of these metabolic disturbances has shown substantial therapeutic potential. For instance, previous studies have established that metformin can alleviate abnormal PASMC proliferation and PAH phenotypes by activating the AMPK pathway. In in vitro and in vivo models, metformin has been shown to significantly activate AMPK signaling, thereby negatively regulating mTOR activity and suppressing aromatase expression, which effectively inhibits PASMC proliferation and reverses pulmonary vascular remodeling [14,15]. Furthermore, a phase II clinical trial in PAH patients revealed Metformin’s ability to improve right ventricular function and reduce lipotoxicity [16]. This evidence collectively suggests that targeting systemic and pulmonary metabolic processes is a promising strategy for PAH. However, Metformin accumulation inhibits mitochondrial complex I and pyruvate carboxylase, leading to a metabolic shift toward massive lactate production [17]. Because intracellular lactate acts as the core substrate for pathogenic histone lactylation in PAH, this metabolic liability may restrict metformin’s utility. Meanwhile, the first dual glucose-dependent insulinotropic polypeptide (GIP) and glucagon-like peptide-1 (GLP-1) receptor agonist, tirzepatide, emerges as a promising alternative. Tirzepatide (TZP) has demonstrated exceptional clinical efficacy in ameliorating insulin resistance, regulating glucose and lipid metabolism, alleviating obesity and reducing the hypoxic burden in patients with OSA [18,19,20,21]. However, whether TZP can act on pulmonary vessels to alleviate PAH and whether the underlying molecular mechanisms involve metabolic and epigenetic remodeling in PASMCs remain unexplored.

Accordingly, this study aims to investigate the protective effects of TZP against PAH and its underlying molecular mechanisms. Utilizing an in vivo monocrotaline (MCT)-induced rat PAH model and in vitro experiments, we found that TZP administration significantly attenuates the synthetic phenotypic switch of PASMCs and ECM remodeling. In addition to modulating the Warburg effect and reducing cellular lactate accumulation, TZP activates the AMPK/SIRT1 signaling axis, thereby reducing the level of histone lactylation at the H4K12 site (H4K12la) in PASMCs. This epigenetic alteration subsequently suppresses the expression of the downstream transcription factor c-Jun and its target genes, which drive the progression of PAH. In summary, this study provides a mechanistic working model highlighting the potential of TZP in attenuating cardiopulmonary vascular diseases by targeting the metabolism-epigenetics axis in PAH.

## 2. Methods

### 2.1. Animals and Treatments

All animal care and experimental protocols were approved by the Institutional Animal Care and Use Committee of Nanjing Drum Tower Hospital (Approval No. 2025AE02033) and conducted in strict compliance with the NIH Guide for the Care and Use of Laboratory Animals. Adult male Sprague-Dawley rats (aged 6–8 weeks, weighing 200–220 g) were purchased from Charles River Laboratories (Beijing, China). All animals were housed in a specific pathogen-free (SPF) facility under a standard 12-h light/dark cycle with ad libitum access to standard rodent chow and water.

To establish the pulmonary arterial hypertension (PAH) model, rats were subjected to a single intraperitoneal injection of monocrotaline (MCT, 60 mg/kg; Sigma-Aldrich, St. Louis, MO, USA). Control rats received an equivalent volume of sterile saline.

For pharmacological and metabolic interventions, rats were randomly assigned to their respective experimental groups. Tirzepatide (TZP; Eli Lilly, Indianapolis, IN, USA) was administered subcutaneously at a dose of 48 ng/g per day for 21 consecutive days. Sodium lactate (NaLac; Aladdin, Shanghai, China) was administered via intraperitoneal injection at a dose of 0.5 g/kg per day for 21 consecutive days. TPA (MedChemExpress, Monmouth Junction, NJ, USA) was administered via intraperitoneal injection at a dose of 50 ng/g per day for 21 consecutive days. At the end of the 21-day induction and intervention period, rats underwent echocardiographic assessments, after which they were euthanized. Blood, lung tissues, and pulmonary arterial trunks were rapidly harvested for subsequent histological and biochemical analyses.

### 2.2. Blood Glucose Measurement

To monitor the systemic metabolic profile during the pharmacological intervention, fasting blood glucose levels were assessed in rats following an overnight fast. The animals were gently restrained, and a minute drop of capillary blood was obtained from the distal tail vein using a sterile lancet. Glucose concentrations were immediately quantified utilizing a handheld portable glucometer (ACCU-CHEK, Roche Diagnostics, Basel, Switzerland) equipped with compatible test strips.

### 2.3. Echocardiography

Transthoracic echocardiography was performed at the 21-day experimental endpoint using a high-resolution micro-ultrasound imaging system (Vevo 2100, VisualSonics, FUJIFILM VisualSonics, Toronto, ON, Canada) equipped with a 30 MHz linear array transducer. Rats were lightly anesthetized with 3.0% isoflurane (R510-22, RWD, Shenzhen, China) and positioned supine on a heated platform to maintain their body temperature at 37 °C. Both heart rate and respiratory rate were continuously monitored throughout the imaging procedure to ensure physiological stability.

To indirectly assess right ventricular afterload and pulmonary arterial changes, pulsed-wave Doppler (PWD) was performed at the parasternal short-axis view at the level of the aortic valve to visualize the right ventricular outflow tract (RVOT) and the main pulmonary artery. The pulmonary artery acceleration time (PAAT) was measured as the time interval from the onset of forward systolic pulmonary flow to peak flow velocity. To evaluate right ventricular (RV) adaptation and structure, right ventricular diameter (RVD) and right ventricular free wall (RVFW) thickness were measured from the parasternal short-axis view using M-mode echocardiography. All echocardiographic measurements were obtained over at least three consecutive cardiac cycles and averaged by a blinded experienced sonographer.

### 2.4. Histological and Immunohistochemical (IHC) Analyses

Lung tissues were fixed in 4% paraformaldehyde for 24 h, followed by dehydration and embedding in paraffin. Serial transverse sections (5 μm) were prepared for histological evaluation. Hematoxylin and Eosin (H&E) staining was performed to assess vascular morphology. Weigert’s elastic staining was performed to visualize the architectural integrity of the elastic fibers.

For IHC staining, paraffin sections (5 μm) were deparaffinized in xylene, rehydrated through a graded ethanol series, and subjected to heat-induced antigen retrieval using EDTA antigen retrieval solution (pH 9.0). The sections were blocked with normal goat serum (31872, ThermoFisher, Waltham, MA, USA) for 1 h at room temperature. Slides were then incubated overnight at 4 °C with a primary antibody against α-SMA (14395-1-AP, Proteintech, Wuhan, China). After rigorous washing with PBS, sections were incubated with the corresponding secondary antibody and visualized using a DAB substrate kit (ab64238, Abcam, Cambridge, UK).

All bright-field histological images were captured using an OLYMPUS (Tokyo, Japan) digital microscope. Quantitative morphometric analysis was strictly performed by two independent investigators blinded to the experimental groups. For each animal, we assessed at least 5 different sections, and a minimum of 20 distal pulmonary arterioles (external diameter < 100 μm) were randomly selected and analyzed using ImageJ software (version 2.16.0). The medial wall thickness (WT%) was calculated using the formula WT% = (2 × medial wall thickness/external diameter) × 100%. The wall area (WA%) was calculated using the formula WA% = (Total vessel area − Lumen area)/Total vessel area × 100%. Pulmonary vascular muscularization was evaluated by categorizing the analyzed arterioles into fully muscularized (M), partially muscularized (P), and non-muscularized (N) vessels based on α-SMA staining. The proportion of each category was calculated as a percentage of the total vessels assessed per animal.

### 2.5. Immunofluorescence (IF) Staining

Tissue IF: Lung sections (5 μm) underwent deparaffinization, rehydration, and heat-induced antigen retrieval as described above. Following permeabilization with 0.3% Triton X-100 (Sigma-Aldrich) in PBS for 15 min, the sections were blocked with normal goat serum (31872, ThermoFisher, Waltham, MA, USA) for 1 h at room temperature. The slides were then incubated overnight at 4 °C with primary antibodies targeting α-SMA (67735, Proteintech, Wuhan, China) and H4K12la (1411RM, PTM BIO, Hangzhou, China).

Cellular IF: Primary PASMCs were seeded on glass coverslips and cultured to the desired confluence. After appropriate treatments, the cells were washed with cold PBS and fixed with 4% paraformaldehyde for 15 min at room temperature. The fixed cells were then permeabilized with 0.1% Triton X-100 (Sigma-Aldrich, St. Louis, MO, USA) for 10 min and blocked with normal goat serum (31872, ThermoFisher, Waltham, MA, USA) for 1 h at room temperature. Subsequently, the cells were incubated overnight at 4 °C with the primary antibody against H4K12la (1411RM, PTM BIO, Hangzhou, China).

Imaging and Quantification: For both tissue and cellular preparations, after rigorous washing with PBS, the samples were incubated with Alexa Fluor-conjugated secondary antibodies (Alexa Fluor 488 and 594, Abcam, Cambridge, UK) for 1 h at room temperature in the dark. Nuclei were counterstained with DAPI (G1012, Servicebio, Wuhan, China). High-resolution fluorescent images were acquired using a confocal scanning microscope (Olympus, Tokyo, Japan).

### 2.6. Primary PASMC Isolation, Culture, and Treatments

Primary rat pulmonary arterial smooth muscle cells (PASMCs) were isolated from the pulmonary arterial trunks of control and MCT-induced PAH rats using a standard enzymatic digestion method. The isolated PASMCs were cultured in Dulbecco’s Modified Eagle Medium (DMEM, Gibco, Waltham, MA, USA) supplemented with 10% fetal bovine serum (FBS, Gibco, Waltham, MA, USA) and 1% penicillin/streptomycin. The cultures were maintained at 37 °C in a humidified incubator with an atmosphere of 5% CO_2_. To avoid spontaneous loss of the contractile phenotype and replicative senescence, only early-passage PASMCs (passages 3–6) were utilized for all in vitro experiments.

For in vitro pharmacological and metabolic interventions, PASMCs were seeded into appropriate culture plates and grown to 70–80% confluence. Cells were then synchronized in serum-free DMEM for 24 h prior to treatments. Depending on the experimental grouping, PASMCs were treated with TZP (6 nM; Eli Lilly, Indianapolis, IN, USA), NaLac (2 mM; Aladdin, Shanghai, China), or the c-Jun activator TPA (20 nM; MedChemExpress, Monmouth Junction, NJ, USA) for 48 h. Control cells were treated with an equivalent volume of the corresponding vehicle.

### 2.7. Cellular Behavior Assays

EdU proliferation assay: Cellular proliferation was evaluated using the 5-ethynyl-2′-deoxyuridine (EdU) incorporation assay kit (KGA9606, KeyGEN BioTECH, Nanjing, China). Briefly, treated PASMCs were incubated with EdU for 2 h, fixed, permeabilized, and stained with Apollo reaction cocktail. Nuclei were counterstained with Hoechst 33342. The proportion of EdU-positive cells was observed under a fluorescence microscope (Axioscope, ZEISS, Oberkochen, Germany) and quantified using ImageJ software (version 2.16.0).

Wound-healing scratch assay: PASMCs were cultured to complete confluence in 12-well plates. A linear scratch was created uniformly across the cell monolayer using a sterile 200 μL pipette tip. After washing with PBS to remove detached cells, the remaining cells were incubated in serum-free medium containing the respective treatments. Images of the scratch wounds were captured at 0 h and 24 h post-scratch, and the wound closure area was analyzed using ImageJ software (version 2.16.0).

Transwell migration assay: Treated PASMCs (1 × 10^4^ cells) suspended in 200 μL of serum-free medium were seeded into the upper Transwell chambers (3422, Corning, Corning, NY, USA), while the lower chambers were filled with 600 μL of complete medium containing 10% FBS. After 48 h of incubation, the cells remaining on the upper surface of the membrane were gently removed with a cotton swab. The cells that migrated to the lower surface were fixed with 4% paraformaldehyde, stained with 0.1% crystal violet, and counted under a light microscope in five randomly selected fields per well. Quantification of migrated cells was conducted by ImageJ software (version 2.16.0).

### 2.8. Cell Transfection

Primary MCT-PASMCs were transfected with small interfering RNA targeting SIRT1 (siSIRT1) or a scrambled negative control siRNA (siNC) (synthesized by HANBIO, Shanghai, China). The transfection was performed utilizing an RNA transfection reagent (jetPRIME, Polyplus, Illkirch, France) strictly according to the manufacturer’s provided protocol. Briefly, PASMCs were seeded in multi-well plates and grown to 50–60% confluence. The siRNA duplexes and the transfection reagent were diluted, mixed and subsequently added dropwise to the cell culture medium. After a 24 h to 48 h incubation period, the knockdown efficiency of SIRT1 was verified by Western blot analysis prior to subsequent pharmacological treatments and downstream functional assays.

For transient overexpression of c-Jun, the full-length rat *Jun* cDNA was subcloned into the pcDNA3.1 expression vector, alongside the corresponding empty vector. Plasmid transfections were conducted using Lipofectamine™ 3000 Transfection Reagent (Invitrogen, Carlsbad, CA, USA) in strict accordance with the manufacturer’s instructions. Briefly, plasmid DNA and P3000™ reagent were pre-diluted in Opti-MEM Reduced Serum Medium (Gibco, Waltham, MA, USA). Lipofectamine 3000 reagent was separately diluted in Opti-MEM. The diluted plasmid-P3000 mixture and Lipofectamine 3000 working solution were gently combined at a 1:1 ratio and incubated for 10–15 min at room temperature. The resulting mixture was added dropwise to the culture medium. Following 6–8 h of incubation at 37 °C in a humidified incubator with 5% CO_2_, the medium was replaced with fresh complete medium. Cells were harvested 24 h to 48 h post-transfection for downstream functional evaluations and immunoblotting to verify overexpression efficiency.

### 2.9. Western Blot Analysis

Total proteins from tissues and primary PASMCs were extracted using ice-cold RIPA lysis buffer (89901, ThermoFisher, Waltham, MA, USA) supplemented with EDTA-free Protease Inhibitor Cocktail (4693132001, Roche, Basel, Switzerland). Tissue samples were mechanically homogenized, whereas cultured PASMCs were directly lysed on ice. After centrifugation (12,000× *g* for 15 min at 4 °C), the protein concentration of the supernatant was quantified utilizing a BCA protein assay kit (P0010, Beyotime, Shanghai, China). Equal amounts of protein extracts were boiled with loading buffer, separated via SDS-PAGE, and transferred onto PVDF membranes (ISEQ00010, Millipore, Billerica, MA, USA). The membranes were incubated in 5% non-fat milk diluted in TBST for 1 h at room temperature, and the blots were then probed overnight at 4 °C with specific primary antibodies [GLP1R (26196, Proteintech, Wuhan, China), GIPR (87123, Proteintech, Wuhan, China), Col1 (72026, Cell Signaling Technology, Danvers, MA, USA), MMP9 (13667, Cell Signaling Technology, Danvers, MA, USA), CNN1 (17819, Cell Signaling Technology, Danvers, MA, USA), α-SMA (14359, Proteintech, Wuhan, China), β-actin (66009, Proteintech, Wuhan, China), GAPDH (60004, Proteintech, Wuhan, China), L-Lactyl-Histone Antibody Sampler Kit (PTM-7093, PTM Bio, Hangzhou, China), HDAC1 (10197, Proteintech, Wuhan, China), HDAC2 (12922, Proteintech, Wuhan, China), HDAC3 (10255, Proteintech, Wuhan, China), SIRT1 (13161, Proteintech, Wuhan, China), SIRT3 (10099, Proteintech, Wuhan, China), p-AMPK (2531, Cell Signaling Technology, Danvers, MA, USA), AMPK (2795, Cell Signaling Technology, Danvers, MA, USA), p-JUN (3270, Cell Signaling Technology, Danvers, MA, USA), c-JUN (9165, Cell Signaling Technology, Danvers, MA, USA), and Cyclin D1 (60186, Proteintech, Wuhan, China)].

After washing steps with TBST, the membranes were incubated with appropriate HRP-conjugated anti-rabbit (7074, Cell Signaling Technology, Danvers, MA, USA) or anti-mouse (7076, Cell Signaling Technology, Danvers, MA, USA) secondary antibodies for 2 h at room temperature. Protein bands were detected utilizing an ECL chemiluminescent substrate (180-501, Tanon, Shanghai, China) and visualized under a digital imaging system (Tanon, Shanghai, China). Protein expression levels were semi-quantified using ImageJ software (version 2.16.0), with β-actin serving as the internal loading control.

### 2.10. Untargeted Metabolomics and Enrichment Analysis

Plasma samples were collected from rats in the MCT and MCT + TZP groups (*n* = 3 per group). The samples were extracted with acetonitrile/methanol and analyzed using a Vanquish UHPLC coupled to a Q Exactive HF-X mass spectrometer (Thermo Fisher Scientific, Waltham, MA, USA). Raw data were processed via XCMS for peak alignment and normalized using unit variance (UV) scaling. Differential metabolites between the MCT and MCT + TZP groups were strictly identified based on a Variable Importance in Projection (VIP) score > 1 (derived from OPLS-DA) and a Student’s *t*-test *p*-value < 0.05.

To investigate the metabolic remodeling mechanisms, comprehensive functional enrichment analyses were performed.

KEGG and HMDB Pathway Enrichment: The identified differential metabolites were annotated and mapped to the KEGG Compound database and the HMDB database. Pathway enrichment was evaluated using hypergeometric tests, and the significantly altered pathways were visualized as bubble plots.

Metabolite Set Enrichment Analysis (MSEA): To capture broader disease-associated metabolic alterations, MSEA was conducted utilizing the MetaboAnalyst platform, mapping our data against established disease-related metabolic sets.

### 2.11. Public Database Mining and Analysis

Public metabolomics data were retrieved from the Metabolomics Workbench repository (Study ID: ST003455), which profiled primary pulmonary arterial smooth muscle cells (PASMCs) from patients with idiopathic pulmonary arterial hypertension (IPAH, *n* = 5) and healthy controls (*n* = 5) using hydrophilic interaction liquid chromatography–mass spectrometry (HILIC-MS) [22]. Raw ion intensities were log2-transformed prior to subsequent statistical analyses.

For targeted quantification, lactate and pyruvate were extracted from the global metabolomic dataset based on RefMet annotations. Group comparisons between IPAH and healthy controls were performed using the Welch’s *t*-test on log2-transformed intensities. Fold change (FC) was calculated as the ratio of group means based on raw intensities.

To visualize the metabolic reprogramming landscape, a targeted panel of 19 metabolites spanning glycolysis, the TCA cycle, and redox cofactors was selected. For heatmap visualization, log2-transformed intensities were z-score normalized across samples within each metabolite. Metabolites were ordered via hierarchical clustering using Euclidean distance and complete linkage. The heatmap was rendered using the seaborn package in Python (version 3.9.6), employing a zero-centered coolwarm diverging colormap.

### 2.12. Seahorse Assay

Real-time metabolic fluxes of primary PASMCs were monitored utilizing a Seahorse XFe96 Extracellular Flux Analyzer (Agilent Technologies, Santa Clara, CA, USA). The oxygen consumption rate (OCR) was measured using the Seahorse XF Cell Mito Stress Test Kit (103015, Agilent, Santa Clara, CA, USA). Briefly, cells were sequentially challenged with oligomycin A (1 μM), carbonyl cyanide-4(trifluoromethoxy)phenylhydrazone (FCCP, 0.5 μM), and a mixture of rotenone and antimycin A (Rot/AA, 0.5 μM). The extracellular acidification rate (ECAR) was quantified via the Seahorse XF Glycolysis Stress Test Kit (103020, Agilent, Santa Clara, CA, USA) through the sequential injection of glucose (10 mM), oligomycin (1 μM) and 2-deoxy-D-glucose (2-DG, 50 mM). All metabolic rates were recorded in real time and subsequently normalized to the total cellular protein content per well to ensure accurate comparative analysis. To ensure statistical rigor, *n* = 6 represents six independent biological replicates, with data from technical replicate wells averaged for each independent biological sample.

### 2.13. RNA-Seq and Transcriptomic Analysis

Total RNA was extracted from primary PASMCs (*n* = 3 independent biological replicates per group) using the TRIzol reagent (Thermo Fisher Scientific, Waltham, MA, USA). RNA concentration and integrity were evaluated using a NanoDrop 2000 (Thermo Fisher Scientific, Waltham, MA, USA) and a LabChip GX system (PerkinElmer, Shelton, CT, USA), respectively, ensuring an RNA Integrity Number (RIN) ≥ 7 for subsequent library construction.

Transcriptome libraries were constructed utilizing the VAHTS Universal V6 RNA-seq Library Prep Kit for Illumina (Vazyme, Nanjing, China). Briefly, mRNA was enriched via magnetic beads, fragmented, and reverse-transcribed into double-stranded cDNA. Following end repair, 3′-adenylation, adapter ligation, and PCR amplification, the products were purified and validated utilizing a Qsep-400 system. Qualified libraries were sequenced on an Illumina NovaSeq X plus platform using a paired-end 150 bp (PE150) strategy.

For bioinformatics analysis, raw reads were filtered using fastp (v0.20.1) to obtain clean data, which were then aligned to the reference genome using HISAT2 (v2.2.1). Transcript assembly and annotation comparison were performed utilizing StringTie (v2.0.4) and gffcompare (v0.9.8), with expression levels quantified as FPKM via the ballgown package. Differential expression analysis was executed using DESeq2 (v1.26.0). Differentially expressed genes (DEGs) were strictly identified based on a *p*-value < 0.05, followed by Gene Ontology (GO) and KEGG pathway enrichment analyses.

### 2.14. Chromatin Immunoprecipitation (ChIP)

ChIP assays were conducted utilizing the SimpleChIP Enzymatic Chromatin IP Kit (9003, Cell Signaling Technology, Danvers, MA, USA) following the manufacturer’s provided protocol. Briefly, PASMCs were fixed with 1% formaldehyde at room temperature to cross-link proteins to DNA, and the reaction was quenched by the addition of glycine. The cells were then harvested, resuspended in Buffer A (7006, Cell Signaling Technology, Danvers, MA, USA), and incubated on ice for 10 min with periodic inverting. After centrifugation (2000× *g* for 5 min at 4 °C), the isolated nuclei were pelleted and resuspended in Buffer B (7007, Cell Signaling Technology, Danvers, MA, USA).

Chromatin fragmentation was achieved through a combination of micrococcal nuclease digestion (0.5 μL, 1 × 10^6^ U/mL) and mild sonication. The sheared chromatin was subsequently subjected to immunoprecipitation overnight at 4 °C using a specific anti-H4K12la antibody (1 μg/μL) or a normal IgG control antibody, followed by incubation with ChIP-grade Protein A/G magnetic beads. After rigorous washing and elution, the cross-links were reversed, and the enriched DNA was purified for subsequent ChIP-seq.

For subsequent high-throughput sequencing, ChIP DNA concentration and quality were assessed utilizing a Qubit 3.0 Fluorometer and an Agilent Bioanalyzer 2100 system. Sequencing libraries were constructed by the Origene Corporation (Chongqing, China) and sequenced on an Illumina platform to generate paired-end reads. Raw reads were filtered using fastp (v0.22.0) to remove low-quality data and adapters. The clean reads were then aligned to the rat reference genome using BWA-MEM (v0.7.17). Peak calling against the input control was performed using MACS2 (v2.2.7.1), with a significance threshold set at q-value < 0.05. The identified peaks were subsequently annotated to their nearest genomic features utilizing the ChIPseeker package (version 1.45.2) in R software (version 4.5.3). Differential peak binding analysis between groups was executed, and peaks with an odds ratio > 2 were defined as significantly differential peaks.

### 2.15. Lactate Level Measurement

Intracellular and serum lactate concentrations were quantitatively determined using a Lactic Acid (LA) Content Assay Kit (BC5340, Solarbio, Beijing, China). Samples (cell lysates or serum) were processed strictly according to the manufacturer’s instructions. To ensure accurate comparisons across different groups, the cell absolute lactate levels were normalized to the corresponding total cellular protein concentrations.

### 2.16. Statistical Analysis

Quantitative data were statistically analyzed and visualized utilizing GraphPad Prism software (version 11.0). All continuous variables are expressed as the mean ± standard deviation (SD). To adhere to the 3Rs principles of animal welfare while maintaining adequate statistical power for the large effect sizes characteristic of the MCT model, the in vivo sample size was established at *n* = 4 independent animals per group. The experimental unit for all statistical tests was defined as the independent animal or biological cell preparation. For measurements involving multiple technical replicates, the values were properly averaged to yield a single mean value per biological replicate prior to statistical group comparisons. For comparisons between two independent groups, an unpaired two-tailed Student’s *t*-test was employed. In cases where equal variance assumptions were violated, Welch’s corrected *t*-test was applied. To evaluate statistical differences among three or more experimental groups, a one-way analysis of variance (ANOVA) was conducted, subsequently followed by Tukey’s post hoc test for multiple comparisons. All in vitro cellular experiments were independently repeated a minimum of three times to ensure reproducibility. A *p*-value of less than 0.05 was considered to indicate a statistically significant difference.

## 3. Results

### 3.1. Tirzepatide Attenuates the Development of the PAH Phenotype and Pulmonary Vascular Remodeling

**Figure 1:**
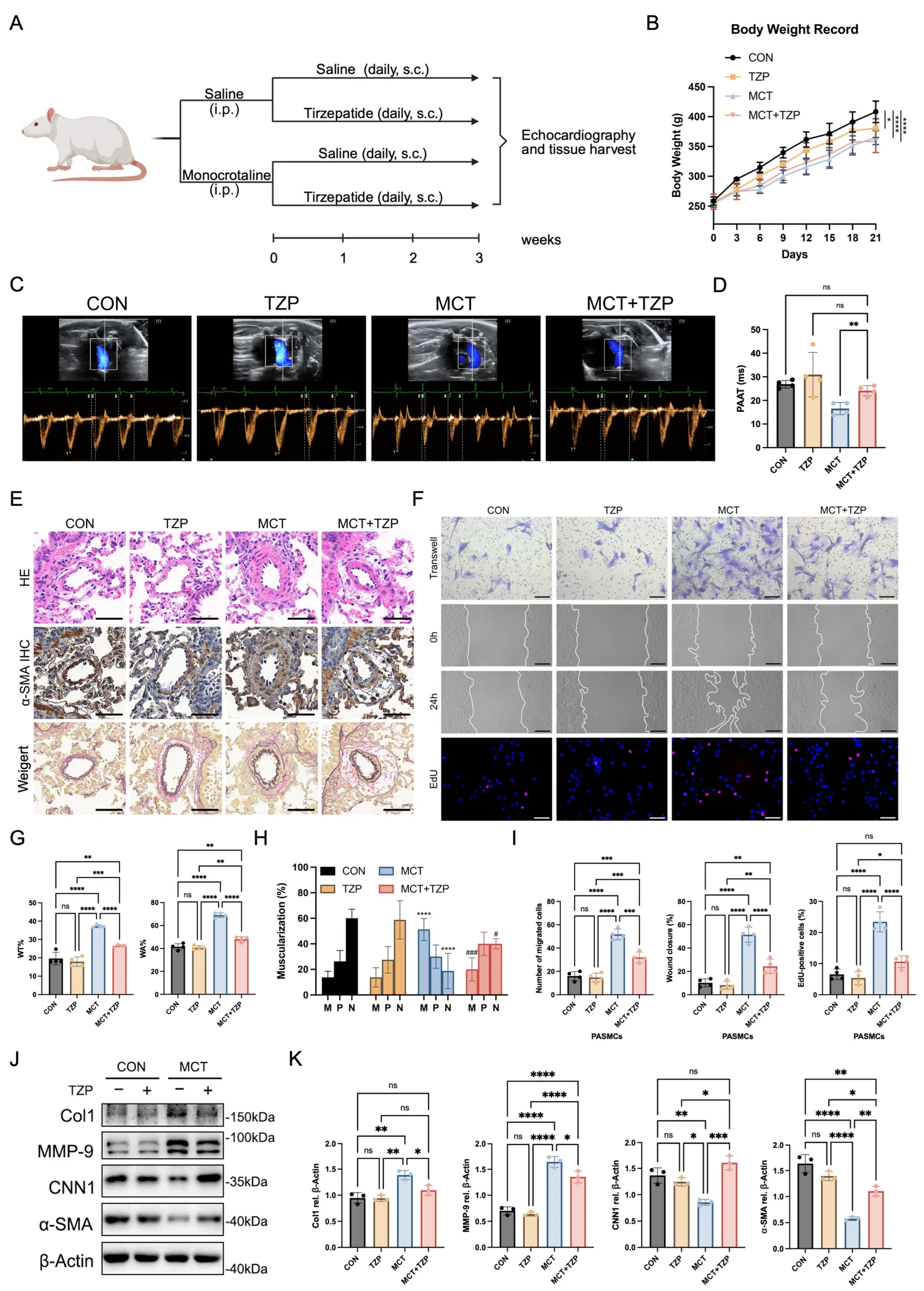
Tirzepatide attenuates the development of the PAH phenotype and pulmonary vascular remodeling. (**A**) Schematic diagram of the experimental design and pharmacological intervention in vivo. (**B**) Longitudinal body weight records during the 21-day administration period (*n* = 4 per group). (**C**) Representative pulmonary artery pulsed-wave Doppler echocardiograms. (**D**) Quantitative analysis of the pulmonary artery acceleration time (PAAT) (*n* = 4 per group). (**E**) Representative images of H&E staining, α-SMA immunohistochemical (IHC) staining, and Weigert’s elastic staining of distal pulmonary arterioles in each group. Scale bars = 50 μm. In α-SMA IHC staining, the brown color indicates α-SMA-positive areas, and the blue color represents cell nuclei. In Weigert’s elastic staining, the dark color indicates elastic fibers, and the pink color represents collagen. (**F**) Representative images of Transwell migration, wound-healing scratch (0 h and 24 h), and EdU incorporation assays in CON or MCT group PASMCs with or without TZP treatment. Scale bars = 100 μm. In EdU assay, red fluorescence represents EdU-positive proliferating cells, and blue fluorescence indicates cell nuclei. (**G**,**H**) Quantitative morphometric analysis of medial wall thickness (WT%) and wall area (WA%) (**G**), and vascular muscularization (**H**) of the distal pulmonary arteries (*n* = 4 per group). (**I**) Corresponding quantitative bar graphs of the Transwell migration, wound closure (%), and EdU-positive cells (%) (*n* = 4 independent biological replicates). (**J**) Representative Western blot images of Col1, MMP-9, CNN1, α-SMA and β-actin levels in CON or MCT group PASMCs with or without TZP treatment. (**K**) Quantitative band density analysis of the corresponding Western blot protein levels (*n* = 3 independent biological replicates). Data are presented as mean ± SD. * *p* < 0.05, ** *p* < 0.01, *** *p* < 0.001, **** *p* < 0.0001 indicate statistical significance between the connected groups; ns = not significant. For (**H**), **** *p* < 0.0001 compared to CON group; # *p* < 0.05, ### *p* < 0.001 compared to MCT group.

To investigate whether tirzepatide (TZP) could attenuate the development of pulmonary arterial hypertension (PAH), we established a rat model of PAH via an intraperitoneal injection of monocrotaline (MCT). The rats were randomly assigned to four groups: healthy control (CON), TZP alone, MCT, and MCT + TZP. In the TZP treatment groups, TZP was administered subcutaneously at a dose of 48 µg/kg/day for 21 days (Figure 1A). Longitudinal body weight tracking showed that MCT exposure reduced weight gain (Figure 1B). While TZP monotherapy slightly decreased the body weight of healthy rats compared to the CON group, it did not further exacerbate the weight loss in MCT-exposed rats (Figure 1B). Blood glucose monitoring during the administration period indicated that TZP did not significantly affect peripheral blood glucose levels in the rats (Supplementary Figure S1A). At the experimental endpoint, echocardiographic assessment revealed that the pulmonary artery acceleration time (PAAT) was significantly shortened in the MCT group compared to the control group, accompanied by the appearance of mid-systolic notching in the pulmonary artery pulsed-wave Doppler flow (Figure 1C,D). To characterize the pulmonary hypertension phenotype and right ventricular (RV) adaptation, we further evaluated RV metrics. MCT-exposed rats developed significant right ventricular hypertrophy and dilation, evidenced by an increased Fulton Index, RV free wall thickness (RVFW), and RV diastolic dimension (RVDd) (Supplementary Figure S1B–E). Notably, TZP treatment markedly attenuated these impairments in pulmonary vascular load and RV remodeling in MCT-exposed rats (Figure 1C,D; Supplementary Figure S1B–E). Furthermore, hematoxylin and eosin (H&E) staining of the endpoint lung tissue sections demonstrated a substantial increase in the medial wall thickness (WT%) and wall area (WA%) of distal pulmonary arteries in the MCT group, which was significantly mitigated by TZP treatment (Figure 1E,G). Immunohistochemical (IHC) staining for α-SMA and Weigert’s elastic stain, alongside the quantification of vascular muscularization, further revealed that TZP intervention effectively attenuated the abnormal smooth muscle hyperproliferation and vascular muscularization observed in the distal pulmonary arterioles of the MCT group (Figure 1E,H). These in vivo findings indicate that TZP exerts a potent protective effect by attenuating the development of MCT-induced PAH, wherein PASMCs play a pivotal role.

To validate these findings in vitro, we isolated primary PASMCs from the pulmonary trunks of the control and MCT-treated rats. Western blot analysis confirmed the protein expression of both the GLP-1 receptor (GLP-1R) and GIP receptor (GIPR) in these isolated PASMCs (Supplementary Figure S1F). The cells were treated with 6 nM of TZP or vehicle for 48 h. Subsequent Transwell migration assays, wound healing assays and EdU proliferation assays demonstrated that TZP significantly inhibited the abnormal proliferation and migration capacities of MCT-PASMCs (Figure 1F,I). Western blot analysis of α-SMA, CNN1, Col1, and MMP9 protein expression levels revealed that MCT stimulation promoted a synthetic phenotypic switch and extracellular matrix (ECM) remodeling, evidenced by the downregulation of contractile markers (α-SMA, CNN1) and the upregulation of Col1 and matrix metalloproteinase MMP9. Strikingly, TZP reversed this phenotypic transition by upregulating α-SMA and CNN1 while simultaneously ameliorating ECM remodeling through the downregulation of Col1 and MMP9 (Figure 1J,K). Taken together, these results suggest that TZP can mitigate MCT-induced PASMC phenotypic switching and ECM remodeling, which likely contribute to the attenuation of the PAH phenotype.

### 3.2. Tirzepatide Ccorrects Warburg Effect to Reduce Glycolysis and Lactate Production In Vivo and in PASMCs

**Figure 2:**
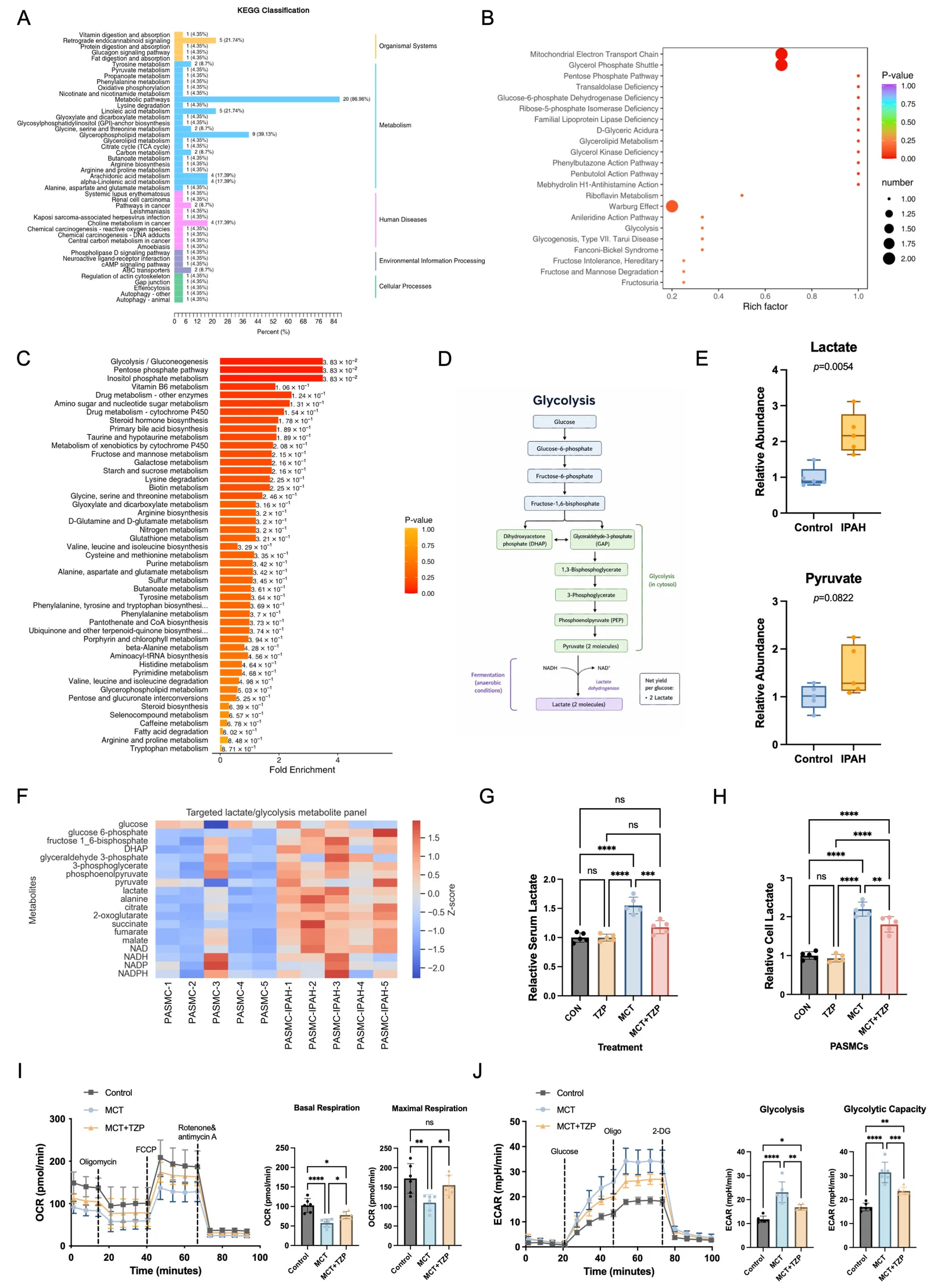
Tirzepatide corrects Warburg effect to reduce glycolysis and lactate production in lung tissues and PASMCs. (**A**) Kyoto Encyclopedia of Genes and Genomes (KEGG) classification of differential metabolites identified by untargeted metabolomics in peripheral blood from the MCT model group versus the MCT + TZP treatment group. (**B**) Bubble scatter plot of KEGG pathway enrichment analysis for the differential metabolites. Color intensity represents the *p*-value, and the size of the dots denotes the number of metabolites mapped to each specific pathway. (**C**) Bar chart illustrating the top enriched KEGG metabolic pathways sorted by fold enrichment. (**D**) Schematic representation of the glycolysis metabolic pathway culminating in lactate generation. (**E**) Box plots comparing the relative abundance of lactate and pyruvate in primary PASMCs derived from healthy controls (*n* = 5 per group) and patients with idiopathic pulmonary arterial hypertension (IPAH, *n* = 5 per group) based on the ST003455 public dataset. (**F**) Heat map of targeted metabolites involved in glycolysis and the tricarboxylic acid (TCA) cycle. Color intensity represents the Z-score normalized relative abundance across samples. (**G**,**H**) Quantitative measurement of relative lactate levels in rat serum (**G**) and in vitro cultured primary PASMCs (**H**) across the designated experimental groups (*n* = 5 per group). (**I**) Oxygen consumption rates (OCRs) of primary PASMCs with indicated treatments, sequentially measured after the addition of oligomycin, FCCP, and rotenone/antimycin A (Rot/AA). Statistical analyses of basal respiration and maximal respiration (*n* = 6 independent biological replicates). (**J**) Extracellular acidification rates (ECARs) of primary PASMCs with indicated treatments, recorded following the sequential addition of glucose, oligomycin (Oligo), and 2-deoxy-D-glucose (2-DG). Statistical analyses of basal glycolysis and glycolytic capacity (*n* = 6 independent biological replicates). Data are expressed as mean ± SD. * *p* < 0.05, ** *p* < 0.01, *** *p* < 0.001, **** *p* < 0.0001, ns = not significant.

To elucidate the underlying mechanisms by which TZP alleviates PAH, we approached the pathology from the perspective of metabolic reprogramming. We performed untargeted metabolomics on peripheral blood samples from the MCT-induced PAH rat model (vehicle-treated) and the MCT + TZP treatment group. KEGG pathway classification revealed that the core differences between the two groups were primarily concentrated in the remodeling of metabolic pathways (Figure 2A). Further KEGG enrichment bubble plots pinpointed the core differential pathways to the Mitochondrial Electron Transport Chain, Warburg Effect, and Glycolysis (Figure 2B). Meanwhile, the fold-enrichment bar chart visually demonstrated that the most significantly enriched pathways were Glycolysis/Gluconeogenesis and its crucial shunt, the Pentose phosphate pathway (Figure 2C). These multi-omics clues strongly suggested that alterations in the glycolytic pathway and the Warburg effect act as the central events in the TZP-mediated alleviation of PAH.

It is well established that during aerobic glycolysis—the hallmark of the Warburg effect—the end products of glucose metabolism are massively converted into lactate by lactate dehydrogenase (Figure 2D). To further clarify the local metabolic characteristics within the pulmonary vasculature, we re-mined and analyzed the ST003455 dataset from the Metabolomics Workbench, an LC-MS metabolomics sequencing cohort comprising PASMCs from five PAH patients and five healthy donors [22]. Although limited by a small sample size, the analysis provided supportive observations that lactate levels in IPAH-PASMCs were significantly higher than those in healthy controls, and the glycolytic intermediate pyruvate also showed an upward trend, though without statistical significance (Figure 2E). Additionally, targeted metabolite heatmaps revealed a comprehensive accumulation of core glycolytic metabolites, including glucose, pyruvate, lactate, and NAD/NADH, in IPAH-PASMCs compared to healthy donor cells (Figure 2F). These findings suggest that cultured human PAH-PASMCs exhibit a pronounced Warburg effect, leading to lactate accumulation.

Guided by these findings, we validated the abnormal accumulation of lactate in vivo and in vitro. By detecting serum lactate concentrations in rats, we found that the overall lactate load was significantly elevated in the MCT model group compared to the healthy control group. However, following TZP treatment, the lactate burden decreased significantly (Figure 2G). In vitro, we measured intracellular lactate content in primary PASMCs from the control and MCT groups following a 48-h treatment with either the vehicle or 6 nM TZP. Consistent with the in vivo data, TZP effectively attenuated MCT-induced intracellular lactate accumulation (Figure 2H).

To obtain definitive evidence of TZP’s regulation of energy metabolic fluxes, we continuously monitored the metabolic profiles of PASMCs across different groups using the Seahorse Extracellular Flux Analyzer. The results showed that TZP-treated MCT-PASMCs exhibited a significant decrease in the extracellular acidification rate (ECAR, indicative of glycolysis) and a concomitant increase in the oxygen consumption rate (OCR, indicative of mitochondrial aerobic respiration) (Figure 2I,J). This metabolic flux data indicates that TZP effectively attenuates the Warburg effect in PAH-PASMCs, redirecting cellular energy metabolism from inefficient, lactate-producing aerobic glycolysis back to the mitochondrial oxidative phosphorylation (OXPHOS) pathway, thereby curtailing the excessive generation and accumulation of lactate.

### 3.3. Lactate Accumulation Aggravates PASMC Phenotypic Switching and Promotes PAH Disease Progression In Vivo and In Vitro

To evaluate the impact of lactate accumulation on PASMC phenotypic switching and PAH disease progression, we conducted forward validation experiments in vivo and in vitro. To avoid potential adverse effects such as systemic acid–base imbalance and non-specific cytotoxicity caused by direct lactic acid supplementation, we selected sodium lactate (NaLac) as the intervention agent to simulate a high-lactate microenvironment.

**Figure 3:**
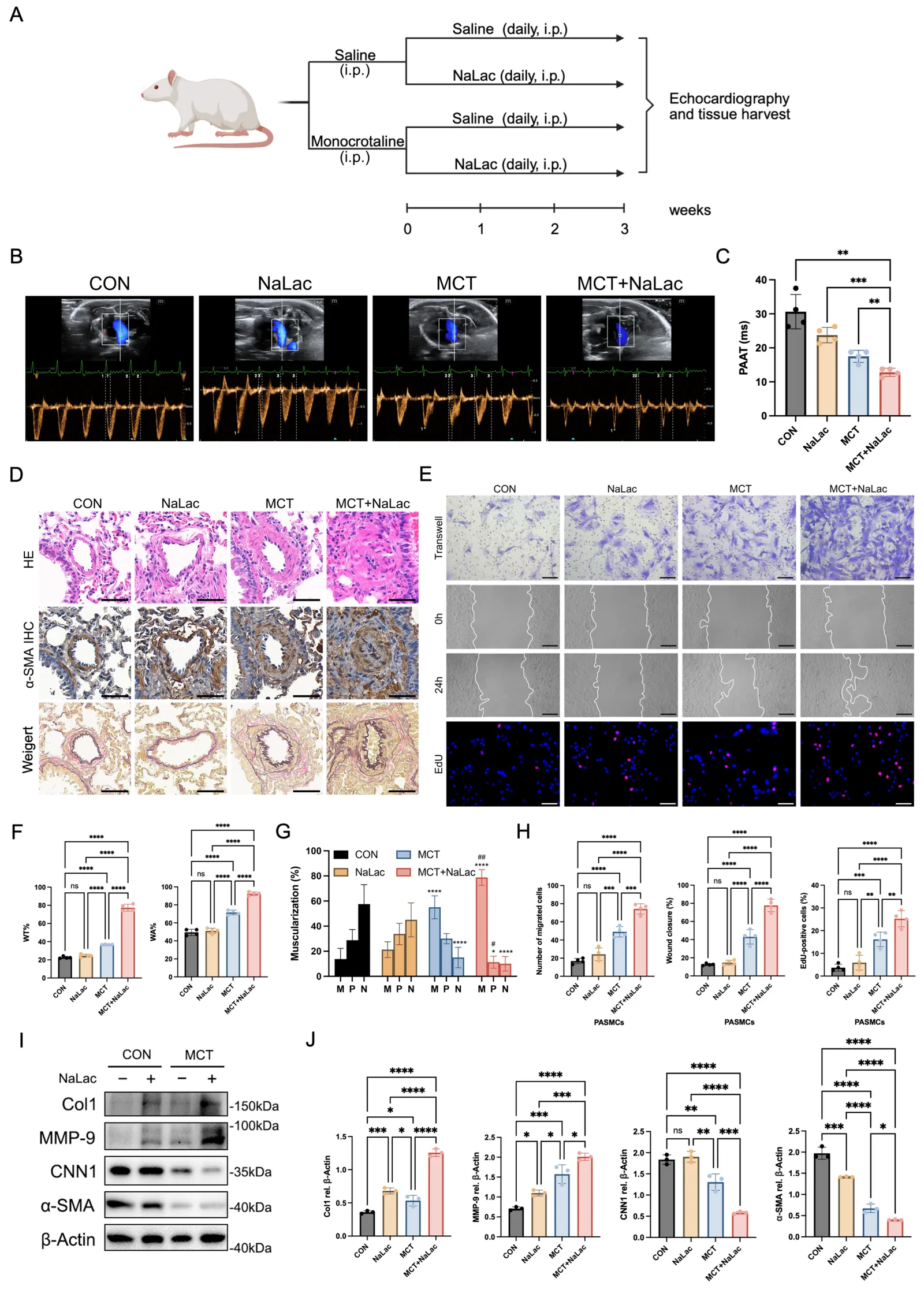
Lactate accumulation aggravates PASMC phenotypic switching and promotes PAH disease progression in vivo and in vitro. (**A**) Schematic diagram of the experimental design and pharmacological intervention in vivo. (**B**) Representative pulmonary artery pulsed-wave Doppler echocardiograms. (**C**) Quantitative analysis of the pulmonary artery acceleration time (PAAT) (*n* = 4 per group). (**D**) Representative images of H&E staining, α-SMA immunohistochemical (IHC) staining, and Weigert’s elastic staining of distal pulmonary arterioles in each group. Scale bars = 50 μm. In α-SMA IHC staining, the brown color indicates α-SMA-positive areas, and the blue color represents cell nuclei. In Weigert’s elastic staining, the dark color indicates elastic fibers, and the pink color represents collagen. (**E**) Representative images of Transwell migration, wound-healing scratch (0 h and 24 h), and EdU incorporation assays in CON or MCT group PASMCs with or without NaLac treatment. Scale bars = 100 μm. In EdU assay, red fluorescence represents EdU-positive proliferating cells, and blue fluorescence indicates cell nuclei. (**F**,**G**) Quantitative morphometric analysis of WT% and WA% (**F**) and vascular muscularization (**G**) of the distal pulmonary arteries (*n* = 4 per group). (**H**) Corresponding quantitative bar graphs of the Transwell migration, wound closure (%) and EdU-positive cells (%) (*n* = 4 independent biological replicates). (**I**) Representative Western blot images of Col1, MMP-9, CNN1, α-SMA and β-actin levels in CON or MCT group PASMCs with or without NaLac treatment. (**J**) Quantitative band density analysis of the corresponding Western blot protein levels (*n* = 3 independent biological replicates). Data are presented as mean ± SD. * *p* < 0.05, ** *p* < 0.01, *** *p* < 0.001, **** *p* < 0.0001 indicate statistical significance between the connected groups; ns = not significant. For (**G**), * *p* < 0.05, **** *p* < 0.0001 compared to CON group; # *p* < 0.05, ## *p* < 0.01 compared to MCT group.

Rats were randomly divided into a healthy control group, a NaLac alone group, an MCT model group, and an MCT + NaLac combined intervention group. NaLac was administered via intraperitoneal injection at a dose of 0.5 g/kg for 21 consecutive days (Figure 3A). Echocardiographic hemodynamic assessment at the endpoint revealed that, building upon the significantly shortened PAAT in the MCT group, the addition of NaLac further aggravated the PAAT shortening and exacerbated the depth of the mid-systolic notching in the pulmonary artery pulsed-wave Doppler profile (Figure 3B,C). RV assessment demonstrated that NaLac treatment significantly worsened MCT-induced RV hypertrophy and dilation, as evidenced by further increases in RVFW, RVDd, and the Fulton Index (Supplementary Figure S2A–C). Subsequent histological analysis and quantitative morphometry demonstrated that the medial wall thickness (WT%) and wall area (WA%) of distal pulmonary arteries were markedly exacerbated in the MCT + NaLac group compared to the MCT group alone (Figure 3D,F). Furthermore, α-SMA IHC staining and Weigert’s elastic fiber staining, along with the quantification of vascular muscularization, confirmed that NaLac significantly intensified the MCT-induced excessive proliferation of smooth muscle cells and vascular muscularization in distal pulmonary arterioles (Figure 3D,G).

In vitro, primary MCT-PASMCs were stimulated with 2 mM NaLac. Transwell, wound healing, and EdU proliferation assays consistently showed that NaLac stimulation remarkably promoted the abnormal proliferation and migration of MCT-PASMCs (Figure 3E,H). Western blot analysis indicated that NaLac stimulation further drove the smooth muscle cells towards a synthetic phenotype and exacerbated ECM remodeling, characterized by a profound downregulation of the contractile markers α-SMA and CNN1, alongside an augmented upregulation of Col1 and MMP9 (Figure 3I,J).

Collectively, these in vivo and in vitro findings demonstrate that lactate accumulation aggravates PASMC synthetic phenotypic switching and PAH progression. These observations support the hypothesis that the metabolic downregulation of lactate accumulation by TZP contributes to its protective effect against PAH progression.

### 3.4. TZP Reduces H4K12la Levels in PASMCs to Attenuate Phenotypic Switching by Activating the AMPK/SIRT1 Axis

**Figure 4:**
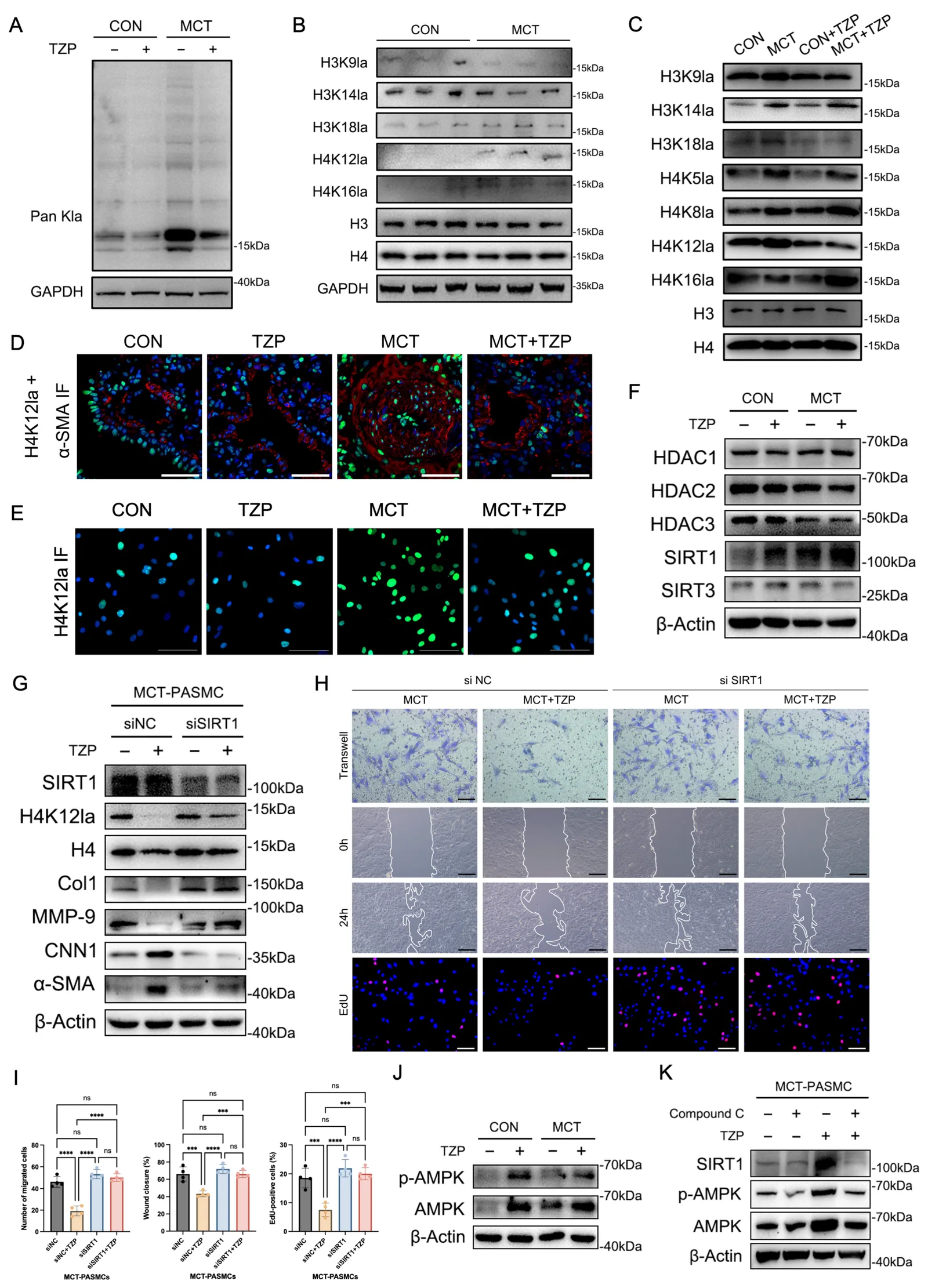
TZP reduces H4K12la levels in PASMCs to attenuate phenotypic switching by activating the AMPK/SIRT1 axis. (**A**) Representative Western blot images of pan-lactylation (Pan Kla) levels in CON or MCT group PASMCs with or without TZP treatment. (**B**) Western blot screening of specific histone lactylation sites and GAPDH in rat pulmonary trunk tissues from the CON and MCT groups. (**C**) Representative Western blot images evaluating the levels of histone lactylation sites and total histones H3 and H4 in CON or MCT group PASMCs with or without TZP treatment. (**D**) Representative dual immunofluorescence staining images of H4K12la and α-SMA in lung tissue sections from each experimental rat group. Green: H4K12la, Red: α-SMA, Blue: DAPI. Scale bars = 50 μm. (**E**) Representative cellular immunofluorescence images of H4K12la in CON or MCT group PASMCs with or without TZP treatment. Green: H4K12la, Blue: DAPI. Scale bars = 100 μm. (**F**) Representative Western blot images screening the expression of HDAC1, HDAC2, HDAC3, SIRT1, and SIRT3 in CON or MCT group PASMCs with or without TZP treatment. (**G**) Representative Western blot images of SIRT1, H4K12la, total H4, Col1, MMP-9, CNN1, α-SMA, and β-Actin levels in MCT-PASMCs transfected with siNC or siSIRT1, in the presence or absence of TZP treatment. (**H**) Representative images of Transwell migration, wound-healing scratch (0 h and 24 h), and EdU incorporation assays in MCT-PASMCs transfected with siNC or siSIRT1, treated with or without TZP. Scale bars = 100 μm. In EdU assay, red fluorescence represents EdU-positive proliferating cells, and blue fluorescence indicates cell nuclei. (**I**) Corresponding quantitative bar graphs of Transwell migration, wound closure (%), and EdU-positive cells (%). (**J**) Representative Western blot images of phosphorylated AMPK (p-AMPK) and total AMPK levels in CON or MCT group PASMCs with or without TZP treatment. (**K**) Representative Western blot images of SIRT1 in MCT-PASMCs treated with DMSO, Compound C, TZP, or Compound C + TZP. Data are presented as mean ± SD. *** *p* < 0.001, **** *p* < 0.0001, ns = not significant.

To further elucidate the specific mechanisms by which TZP-mediated downregulation of lactate metabolism attenuates PASMC phenotypic remodeling, we assessed pan-lactylation levels via Western blot in CON-PASMCs, CON-PASMCs + TZP, MCT-PASMCs and MCT-PASMCs + TZP. We found that MCT-PASMCs exhibited higher global histone lactylation levels compared to CON-PASMCs, and this was notably attenuated by TZP (Figure 4A). Through targeted screening of specific histone lactylation sites in rat pulmonary trunk tissues and PASMC protein samples, we identified histone H4 lysine 12 lactylation (H4K12la) as a critical target. H4K12la levels were significantly elevated in both the pulmonary trunks of PAH rats and primary PASMCs, while TZP treatment effectively suppressed H4K12la levels in MCT-PASMCs (Figure 4B,C; corresponding quantitative band density analyses are provided in Supplementary Figure S3A,B). To validate this specific modification, we performed dual immunofluorescence staining for α-SMA and H4K12la on lung tissue sections. The results revealed an intense enrichment of H4K12la signals within the pulmonary arterioles of the MCT model rats, which was significantly inhibited by TZP intervention (Figure 4D). Consistently, in vitro immunofluorescence staining of PASMCs revealed that the high H4K12la signals in MCT-PASMCs were markedly suppressed by TZP (Figure 4E).

To clarify the mechanism underlying TZP’s regulation of H4K12la, we screened several delactylases, including HDAC1, HDAC2, HDAC3, SIRT1, and SIRT3, via Western blot. We observed a specific upregulation of SIRT1 in MCT-PASMCs following TZP treatment (Figure 4F, Supplementary Figure S3C). To further verify whether TZP downregulates H4K12la modifications via SIRT1, we knocked down SIRT1 expression in MCT-PASMCs using siRNA transfection. Western blot results indicated that the H4K12la-suppressing capability of TZP was significantly blunted following SIRT1 silencing (Figure 4G, Supplementary Figure S3D). Correspondingly, TZP’s regulatory effects on the phenotypic markers were also substantially weakened, including the upregulation of α-SMA and CNN1 and the downregulation of Col1 and MMP9 (Figure 4G, Supplementary Figure S3D). Transwell, wound healing, and EdU proliferation assays further demonstrated that SIRT1 knockdown markedly impaired TZP’s ability to inhibit the abnormal proliferation and migration behaviors of MCT-PASMCs (Figure 4H,I).

It is widely recognized that TZP facilitates the shift from anaerobic glycolysis toward oxidative phosphorylation. Through WB analysis of the PASMC protein samples, we observed that AMPK, a key enzyme regulating oxidative phosphorylation, was significantly activated and upregulated in the TZP-treated MCT-PASMCs (Figure 4J, Supplementary Figure S3E). Given that SIRT1 acts as a direct downstream target of AMPK, we utilized Compound C, a selective AMPK inhibitor, to confirm this mechanistic link. The results demonstrated that co-treatment with Compound C reduced the TZP-induced upregulation of SIRT1 (Figure 4K, Supplementary Figure S3F), indicating that TZP upregulates SIRT1 expression in an AMPK-dependent manner.

Together, these findings suggest that TZP attenuates H4K12 lactylation by activating the AMPK/SIRT1 axis, thereby inhibiting the abnormal migration and proliferation of PASMCs and alleviating PAH.

### 3.5. TZP Inhibits H4K12la-Mediated Transcription of the Proto-Oncogene Jun in PASMCs

**Figure 5:**
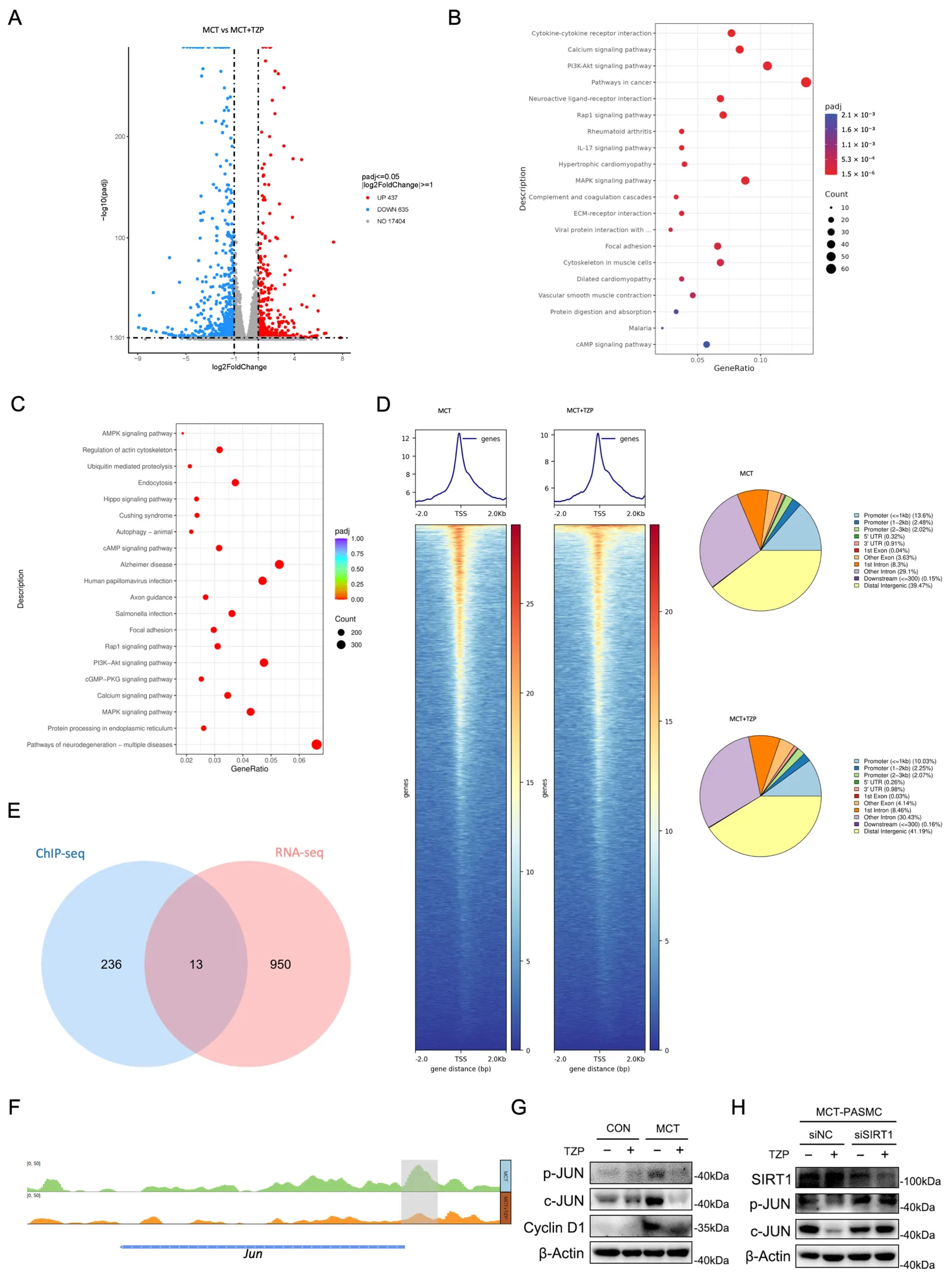
TZP inhibits H4K12la-mediated transcription of the proto-oncogene *Jun* in PASMCs. (**A**) Volcano plot of differentially expressed genes identified by RNA-seq in PASMCs between the MCT and MCT + TZP groups. (**B**) KEGG pathway enrichment analysis of the differentially expressed genes from RNA-seq in PASMCs between the MCT and MCT + TZP groups. (**C**) KEGG pathway enrichment analysis of the target genes with significant alterations in H4K12la binding derived from ChIP-seq. (**D**) Heatmap and average profile of H4K12la enrichment around the transcription start sites (TSSs), alongside pie charts illustrating the genomic feature distribution of H4K12la peaks in MCT and MCT + TZP PASMCs. (**E**) Venn diagram illustrating the intersection of differential genes identified by both RNA-seq and ChIP-seq. (**F**) Integrative Genomics Viewer (IGV) tracks visualizing the H4K12la binding peaks at the *Jun* promoter region in the MCT and MCT + TZP groups. The shaded area highlights the reduced H4K12la enrichment. (**G**) Representative Western blot images of phosphorylated c-Jun (p-JUN), total c-JUN, Cyclin D1, and β-Actin in CON or MCT group PASMCs with or without TZP treatment. (**H**) Representative Western blot images of SIRT1, p-JUN, c-JUN, and β-Actin in MCT-PASMCs transfected with siNC or siSIRT1, in the presence or absence of TZP treatment.

To identify the downstream effector molecules responsible for mediating the TZP-induced suppression of H4K12la and subsequent attenuation of PASMC phenotypic switching, we performed transcriptomic sequencing (RNA-seq) and chromatin immunoprecipitation sequencing (ChIP-seq) on MCT-PASMCs and TZP-treated MCT-PASMCs. The volcano plot of differential genes derived from RNA-seq (Figure 5A) and the subsequent KEGG pathway enrichment analysis revealed that the significantly altered transcripts were predominantly enriched in pathways closely associated with hyperactive cellular proliferation, such as the PI3K-AKT and MAPK signaling pathways (Figure 5B). Correspondingly, the KEGG analysis of ChIP-seq data also mapped the target genes with significant alterations in H4K12la modification to core pathways, including PI3K-AKT and MAPK (Figure 5C).

Subsequently, we deeply analyzed the genome-wide binding profile of H4K12la using ChIP-seq data. The enrichment heatmap and signal distribution profile demonstrated that H4K12la signals were highly concentrated around the transcription start sites (TSSs) of genes. Notably, TZP intervention globally diminished the binding abundance of H4K12la at these TSS regions (Figure 5D). To precisely pinpoint the key target genes, we performed a combined intersection analysis of the differential results from RNA-seq and ChIP-seq, eventually identifying a subset of 13 core overlapping genes exhibiting synchronized alterations in both transcriptional expression and H4K12la modification levels (Figure 5E). The full list of these 13 genes is provided in Supplementary Table S1. Among these candidates, the proto-oncogene *Jun* (encoding the c-Jun protein) exhibited the most statistically significant modification change in the CUT and Tag dataset. As a master transcription factor and a core component of the AP-1 complex, c-Jun classically drives cellular hyperproliferation and migration. The Integrative Genomics Viewer (IGV) tracks from ChIP-seq confirmed a high H4K12la binding peak at the promoter region of the *Jun* gene in the MCT group, whereas the addition of TZP significantly suppressed this H4K12la enrichment (Figure 5F). Quantitative real-time PCR (qPCR) validation confirmed that *Jun* transcription is significantly upregulated in MCT-PASMCs, which is markedly suppressed by TZP treatment (Supplementary Figure S4A). More importantly, direct supplementation with exogenous lactate (MCT + NaLac group) further exacerbated *Jun* transcription (Supplementary Figure S4A). Consistently, Western blot analysis demonstrated that the levels of H4K12la, alongside the phosphorylation and total protein expression of c-Jun, were dose-dependently elevated following NaLac stimulation (Supplementary Figure S4B,C). This suggests that TZP suppresses *Jun* transcription by removing the lactylation marks at its promoter region.

To validate the crucial role of c-Jun and its downstream signaling in PASMC phenotypic switching at the protein level, we evaluated the phosphorylation signals and total protein expression of c-Jun in CON, TZP, MCT, and MCT + TZP PASMCs using Western blot. The results showed that both the phosphorylation and total protein levels of c-Jun were aberrantly upregulated in MCT-PASMCs, and this was diminished by TZP (Figure 5G, Supplementary Figure S4C). Furthermore, we examined Cyclin D1, a classic downstream pro-cell-cycle target of c-Jun, and found its expression trend was identical to that of c-Jun, indicating that c-Jun drives the proliferative phenotype of PASMCs through Cyclin D1 regulation (Figure 5G, Supplementary Figure S4C). Additionally, the existing literature demonstrates that c-Jun can promote the expression of MMPs [23,24,25], which aligns with our earlier finding (Figure 1J) that TZP downregulates MMP9 expression and attenuates PASMC migratory capacity. Moreover, Western blot analysis revealed that silencing SIRT1 in MCT-PASMCs effectively abrogated the suppressive effects of TZP on both the phosphorylation and total protein expression of c-Jun (Figure 5H, Supplementary Figure S4D).

In summary, these results elucidate that TZP activates the AMPK/SIRT1 axis, thereby downregulating histone H4K12 lactylation levels and diminishing H4K12la enrichment at the *Jun* promoter. This epigenetic suppression inhibits c-Jun transcription and activation, subsequently downregulating downstream pro-proliferative (Cyclin D1) and pro-migratory (MMP9) factors, ultimately attenuating PASMC phenotypic switching and PAH progression.

### 3.6. Pharmacological Activation or Genetic Overexpression of c-Jun Counteracts the Therapeutic Benefits of TZP in PASMC Phenotypic Switching and PAH

**Figure 6:**
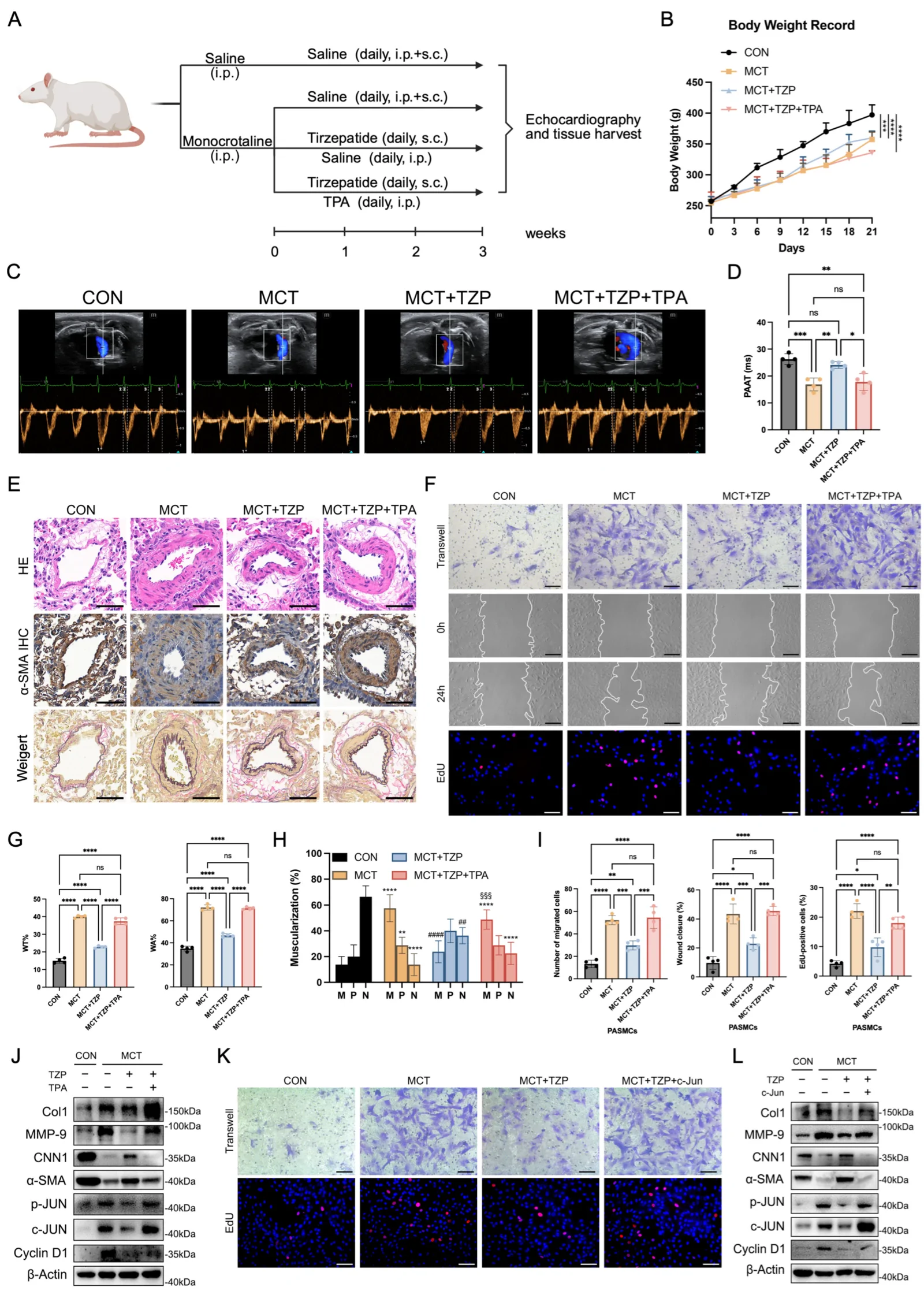
Pharmacological activation or genetic overexpression of c-Jun counteracts the therapeutic benefits of TZP in PASMC phenotypic switching and PAH. (**A**) Schematic diagram of the experimental design and pharmacological intervention in vivo. (**B**) Longitudinal body weight records during the 21-day administration period (*n* = 4 per group). (**C**) Representative pulmonary artery pulsed-wave Doppler echocardiograms. (**D**) Quantitative analysis of the pulmonary artery acceleration time (PAAT) (*n* = 4). (**E**) Representative images of H&E staining, α-SMA immunohistochemical (IHC) staining, and Weigert’s elastic staining of distal pulmonary arterioles in each group. Scale bars = 100 μm. In α-SMA IHC staining, the brown color indicates α-SMA-positive areas, and the blue color represents cell nuclei. In Weigert’s elastic staining, the dark color indicates elastic fibers, and the pink color represents collagen. (**F**) Representative images of Transwell migration, wound-healing scratch (0 h and 24 h), and EdU incorporation assays in CON/MCT group PASMCs, MCT group PASMCs with TZP treatment or MCT group PASMCs with combined TZP and TPA treatment. Scale bars = 100 μm. In EdU assay, red fluorescence represents EdU-positive proliferating cells, and blue fluorescence indicates cell nuclei. (**G**,**H**) Quantitative morphometric analysis of medial wall thickness (WT%) and wall area (WA%) (**G**), and vascular muscularization (**H**) of the distal pulmonary arteries (*n* = 4 per group). (**I**) Corresponding quantitative bar graphs of the Transwell migration, wound closure (%), and EdU-positive cells (%) (*n* = 4 independent biological replicates). (**J**) Representative Western blot images of Col1, MMP-9, CNN1, α-SMA, p-JUN, c-JUN, Cyclin D1 and β-actin levels in CON/MCT group PASMCs, MCT group PASMCs with TZP treatment or MCT group PASMCs with combined TZP and TPA treatment. (**K**) Representative images of Transwell migration and EdU incorporation assays in MCT group PASMCs treated with TZP in the presence or absence of c-Jun overexpression plasmid. Scale bars = 100 μm. In EdU assay, red fluorescence represents EdU-positive proliferating cells, and blue fluorescence indicates cell nuclei. (**L**) Representative Western blot images of Col1, MMP-9, CNN1, α-SMA, p-Jun, c-Jun, Cyclin D1, and β-actin levels in MCT group PASMCs treated with TZP in the presence or absence of c-Jun overexpression plasmid. Data are presented as mean ± SD. * *p* < 0.05, ** *p* < 0.01, *** *p* < 0.001, **** *p* < 0.0001, ns = not significant. For (**H**), ** *p* < 0.01, **** *p* < 0.0001 compared to CON group; ## *p* < 0.01, #### *p* < 0.0001 compared to MCT group; §§§ *p* < 0.001 compared to MCT + TZP group.

To verify whether c-Jun serves as a critical downstream target mediating the therapeutic efficacy of TZP against PAH, we utilized TPA, a c-Jun activator, to conduct pharmacological rescue experiments both in vivo and in vitro. In vivo, rats were randomly assigned to four groups: healthy control (CON), MCT model, MCT + TZP treatment, and MCT + TZP + TPA combined treatment, with TPA administered intraperitoneally at a dose of 50 µg/kg for 21 days (Figure 6A). Throughout the experiment, blood glucose levels remained stable across all groups (Supplementary Figure S5A), and TZP did not further exacerbate the body weight loss in MCT-induced rats (Figure 6B).

Echocardiographic hemodynamic assessments at the endpoint revealed that, contrasting with the significantly prolonged PAAT achieved by TZP intervention, the addition of TPA caused a re-shortening of PAAT and exacerbated the depth of mid-systolic notching in the pulmonary artery pulsed-wave Doppler profile (Figure 6C,D). RV assessment demonstrated that TPA treatment effectively negated the protective effects of TZP against RV hypertrophy and dilation, as evidenced by the increases in RVFW, RVDd, and the Fulton Index (Supplementary Figure S5B–E). Subsequent histological analysis and quantitative morphometry demonstrated that the abnormal medial wall thickness (WT%) and wall area (WA%) of distal pulmonary arteries were markedly re-exacerbated in the MCT + TZP + TPA group compared to the MCT + TZP group (Figure 6E,G). Furthermore, α-SMA IHC staining and Weigert’s elastic fiber staining, along with the quantification of vascular muscularization, demonstrated that TPA significantly blunted the mitigating effects of TZP on the excessive proliferation of smooth muscle cells and vascular muscularization in distal pulmonary arterioles (Figure 6E,H).

To validate this mechanism in vitro, we intervened in MCT-PASMCs using both the pharmacological activator TPA (20 nM) and genetic c-Jun overexpression plasmids. EdU proliferation, Transwell migration, and wound healing assays consistently demonstrated that the pharmacological activation of c-Jun weakened the inhibitory constraints imposed by TZP, enabling MCT-PASMCs to regain highly active abnormal proliferation and migration capabilities (Figure 6F,I). Crucially, parallel functional assays utilizing c-Jun overexpression yielded similar phenotypic counteractions (Figure 6K, Supplementary Figure S5H). Subsequent Western blot and quantitative analysis revealed that the combined TZP and TPA treatment re-promoted the transition of smooth muscle cells toward a synthetic phenotype and re-initiated ECM remodeling, which was characterized by the renewed downregulation of α-SMA/CNN1 and the upregulation of Col1/MMP9 (Figure 6J, Supplementary Figure S5F). Similar protein expression trends were independently recapitulated in the genetic c-Jun overexpression model (Figure 6L, Supplementary Figure S5G).

In conclusion, these in vivo and in vitro rescue experiments demonstrate that the activation or overexpression of the c-Jun signaling pathway counteracts the protective effects of TZP on PAH. This evidence further verified the anti-PAH efficacy of TZP is at least partially achieved by targeting and inhibiting the abnormal histone lactylation (H4K12la), thereby downregulating the expression and activation of the downstream gene *Jun*, which ultimately curtails the abnormal proliferation, migration, and phenotypic switching of PASMCs.

## 4. Discussion

In the present study, we identified the potential protective effect of Tirzepatide (TZP), a novel dual GIP and GLP-1 receptor agonist, on PASMC phenotypic switching and the progression of PAH. Our mechanistic investigations revealed that TZP intervention leads to profound metabolic and epigenetic remodeling in PAH-PASMCs. Specifically, TZP treatment suppresses aerobic glycolysis and lactate accumulation, activates the AMPK/SIRT1 signaling axis, and consequently diminishes pathogenic histone H4 lysine 12 lactylation along with its downstream target, c-Jun. Downregulation of c-Jun contributes to subsequent reduction of its downstream effectors, including MMP9 and Cyclin D1, thereby inhibiting aberrant PASMC proliferation and migration. These findings highlight the possibility of exploring TZP’s therapeutic applications beyond metabolic syndromes, positioning it as a promising mechanism-targeted pharmacological strategy for PAH.

The progressive occlusion of pulmonary arterioles, driven primarily by aberrant pulmonary vascular remodeling, represents the therapeutic challenge in managing PAH. At the cellular level, this structural deterioration is largely sustained by the phenotypic switching of PASMCs, wherein cells lose their contractile state and switch into a hyperproliferative and synthetic phenotype [26,27]. This chronic cellular drift leads to continuous thickening and eventual right ventricular failure [28]. Our in vivo and in vitro evaluations demonstrate that TZP administration counteracts this pathogenic transformation, effectively preserving the contractile apparatus of PASMCs. Instead of merely dilating blood vessels to temporarily relieve symptoms, TZP intervenes in the underlying cellular changes that cause vascular remodeling, proving its potential to fundamentally alter the disease progression.

Mechanistically, the aberrant phenotypic switching and hyperproliferation of PASMCs are partially promoted by the Warburg effect. Within this highly glycolytic microenvironment, the accumulating lactate can act as an epigenetic substrate for lysine lactylation. Since its initial discovery, histone lactylation has emerged as a fundamental epigenetic mechanism dictating transcriptional profiles and cellular behaviors across various pathological conditions [29,30,31]. Emerging evidence validates its particularly critical role in cardiovascular diseases, especially in driving aberrant vascular smooth muscle cell (VSMC) phenotypic switching [8]. In this study, we observed a positive feedback loop between metabolic reprogramming, lactate accumulation and gene expression: the metabolic shift of PASMCs toward aerobic glycolysis leads to intracellular lactate accumulation, which subsequently promotes histone H4 lysine 12 lactylation (H4K12la) and transcriptional expression of c-Jun. Notably, c-Jun, as a core subunit of the AP-1 transcription complex, is known to hyperactivate aerobic glycolysis by upregulating several key glycolytic enzymes, such as hexokinase and pyruvate kinase, either through direct transcriptional activation or via downstream metabolic cascades [32,33,34]. This implies that c-Jun hyperactivation worsens the metabolic environment by shifting energy production toward glycolysis, which produces excessive lactate and consequently creates a vicious cycle. The highlight of this study is that Tirzepatide not only improves global metabolic homeostasis but also disrupts this positive feedback loop by upregulating the AMPK/SIRT1 axis to downregulate H4K12la, ultimately remodeling both the metabolism and phenotype of PASMCs.

Although previous studies have demonstrated that metformin can alleviate PAH by activating the AMPK pathway, we selected Tirzepatide as the primary interventional agent in this study due to its superiority in energy metabolic remodeling and potential clinical safety [35,36]. Metformin takes effect by inhibiting mitochondrial respiratory chain complex I, which induces a decrease in the intracellular ATP/AMP ratio and passively activates AMPK [37]. However, this suppression of mitochondrial function inevitably forces cells to compensatorily rely on anaerobic glycolysis, posing a potential risk of inducing hyperlactatemia or lactic acidosis [17]. For PAH patients who already suffer from systemic hypoxia and abnormal lactate accumulation in the pulmonary environment, this represents a pharmacological hazard. Conversely, TZP actively mediates the activation of downstream signaling pathways by specifically binding to GLP-1R or GIPR. Our Seahorse metabolic flux assay confirmed that TZP treatment does not impair mitochondrial function but effectively corrects the Warburg effect in PAH-PASMCs, redirecting energy metabolism from inefficient glycolysis back to mitochondrial oxidative phosphorylation (OXPHOS). This metabolic reprogramming intercepts the conversion of pyruvate to lactate at the source, thereby substantially reducing the total intracellular lactate burden. Beyond these direct effects on PASMCs, the systemic effects of TZP treatment should not be overlooked. By dual-targeting GLP-1 and GIP receptors throughout the body, TZP confers profound whole-body metabolic benefits, including the improvement of peripheral insulin sensitivity, suppression of circulating pro-inflammatory cytokines, and optimization of cardiovascular hemodynamics [38,39,40,41]. These systemic actions may function cooperatively with local pulmonary vascular mechanisms, collectively contributing to the retardation of disease progression and the mitigation of secondary cardiac stress.

Furthermore, this study provides novel insights into how TZP reduces histone lactylation marks. We found that TZP can activate AMPK and subsequently upregulate downstream SIRT1 expression. While our data highlight the significant role of the AMPK/SIRT1 axis, TZP may enhance SIRT1 via multiple parallel pathways. For instance, classic receptor activation intrinsically triggers the cAMP/PKA signaling pathway, which can directly transactivate the SIRT1 promoter [42,43]. Additionally, the TZP-driven restoration of mitochondrial OXPHOS can elevate the intracellular NAD+/NADH ratio, which consequently boosts SIRT1 catalytic activity [44,45,46]. Recent breakthrough studies have established that SIRT1 is not only a canonical deacetylase but also possesses potent delactylase activity [47,48,49]. Therefore, TZP mediates a synergistic regulatory effect: on one hand, it diminishes de novo lactate production by restoring OXPHOS; on the other hand, it actively downregulates established H4K12 lactylation via SIRT1 upregulation. Nonetheless, further direct biochemical investigations, such as in vitro recombinant SIRT1 delactylase activity assays and kinetic analyses, are warranted to conclusively determine its precise catalytic contribution to site-specific H4K12 delactylation. Multiple large-scale clinical trials have already validated the comprehensive benefits of TZP in ameliorating cardiovascular metabolic syndromes, such as obesity and insulin resistance, both of which are prominent risk factors for PAH [39]. This study provides preliminary mechanistic evidence supporting the potential of TZP as an attractive, metabolism-targeted therapeutic strategy worthy of further translational investigation in PAH.

Several limitations and open questions in the current study warrant thoughtful consideration. Although we localized the AMPK/SIRT1/H4K12la/c-Jun axis within PASMCs, PAH pathogenesis is driven by complex intercellular crosstalk. Recent studies have clarified that substantial lactate excretion resulting from highly glycolytic synthetic smooth muscle cells can alter the local microenvironment, promoting histone lactylation and the phenotypic switch of neighboring cells, thereby facilitating the expansion of the diseased area [8]. The potential modulatory effects of TZP on this lactate-mediated alteration, as well as its interactions with neighboring endothelial cells and macrophages, remain an open question. Additionally, while in vitro plasmid transfection substantiated the isolated role of c-Jun overexpression, TPA functions as a pan-activator across the AP-1 complex. Considering the current lack of selective small-molecule c-Jun activators, subsequent in vivo investigations employing targeted viral vectors are warranted to further dissect its unconfounded actions in pulmonary vascular remodeling. In addition, future studies utilizing smooth muscle-specific GLP-1R/GIPR knockout murine models are necessary to precisely decouple these direct pulmonary vascular actions from secondary systemic benefits. Finally, potential preclinical constraints regarding the animal cohort should be noted. Our in vivo evaluations were conducted with a relatively modest sample size and utilized exclusively male rats. Given the well-established sexual dimorphism in PAH pathogenesis, alongside potential sex-divergent responses in GLP-1/GIP-mediated metabolic signaling, future studies enrolling both female cohorts and expanded sample sizes across diverse preclinical models will be indispensable to fully assess the generalizability and translational promise of TZP.

## 5. Conclusions

Our study demonstrates that metabolic reprogramming towards the Warburg effect and the consequent intracellular lactate accumulation serve as critical drivers of PASMC phenotypic switching and pulmonary vascular remodeling in PAH. Building upon this pathogenic foundation, we demonstrated that Tirzepatide administration effectively alleviates PAH by simultaneously regulating the AMPK/SIRT1/H4K12la/c-Jun axis and correcting this metabolic derangement. Ultimately, our work highlights the critical role of histone lactylation in governing vascular cell behavior and disease progression and offers translational relevance for TZP as a potential preventive and disease-modifying strategy in PAH.

## Data Availability

The raw data supporting the conclusions of this article will be made available by the authors on request.

## Supplementary Materials

The following supporting information can be downloaded at: https://www.mdpi.com/article/10.3390/cells15201900/s1, Figure S1: The effects of Tirzepatide on systemic blood glucose and right ventricular remodeling in MCT-induced PAH rats; Figure S2: The effects of exogenous lactate on right ventricular remodeling in MCT-induced PAH rats; Figure S3: Quantitative densitometric analysis of Figure 4B, C, F, G, J, K; Figure S4: Independent validation of lactate-driven *Jun* transcription, dose-dependent induction by NaLac, and quantitative densitometric analysis of Figure 5G, H; Figure S5: Evaluation of systemic glycemic stability, right ventricular remodeling, and quantitative analyses of pharmacological and genetic c-Jun rescue experiments; Table S1: 13 core overlapping genes exhibiting synchronized alterations in both transcriptional expression and H4K12la modification levels.

## Abbreviations

AMPK, AMP-activated protein kinase; GIP, glucose-dependent insulinotropic polypeptide; GLP-1, glucagon-like peptide-1; H4K12la, H4 lysine 12 lactylation; MCT, monocrotaline; NaLac, Sodium lactate; PASMCs, pulmonary arterial smooth muscle cells; PAH, pulmonary arterial hypertension; SIRT1, Sirtuin 1; TPA, 12-O-Tetradecanoylphorbol-13-Acetate; TZP, Tirzepatide.
