# Supplementary material for: Tirzepatide Attenuates Pulmonary Vascular Remodeling in Pulmonary Arterial Hypertension: Association with the SIRT1/H4K12la/c-Jun Axis
Source: Cells. 2026 Oct 19;15(20):1900. doi: 10.3390/cells15201900 (PMC13702887; doi:10.3390/cells15201900)
Supplement: Supplementary file 1 [file cells-15-01900-s001.zip › supplementary files/Supplementary Figure.pdf]

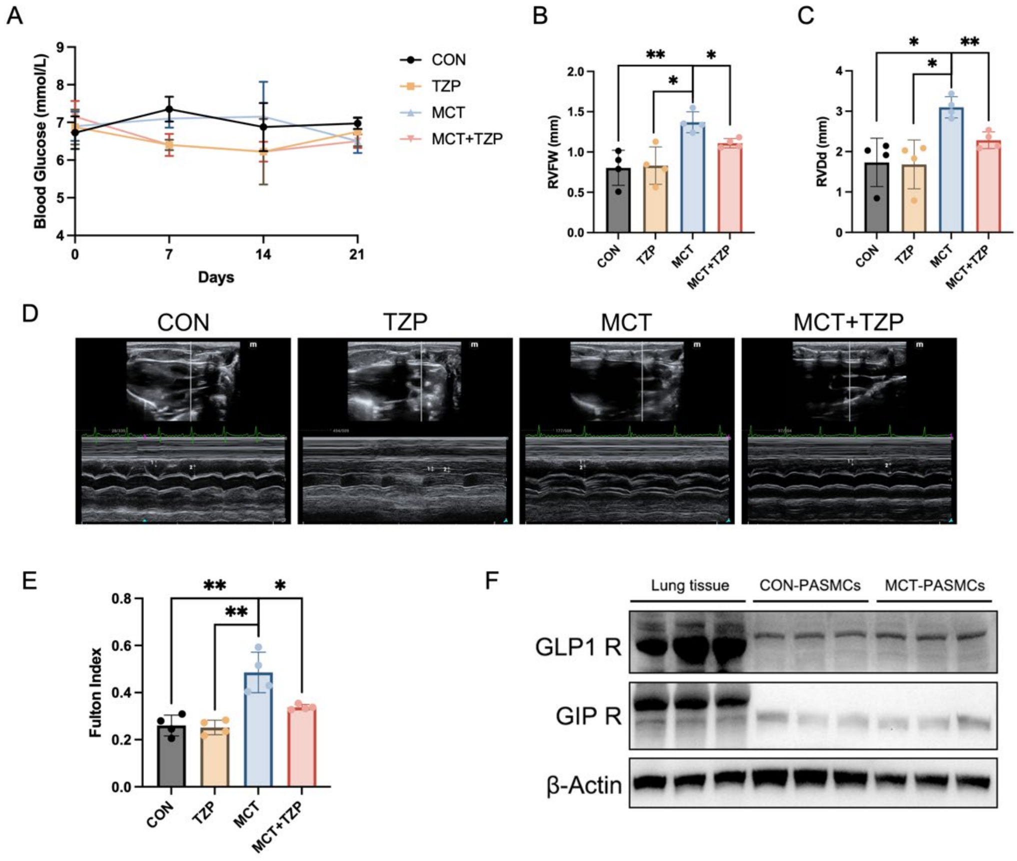

Supplementary Figure S1. The effects of Tirzepatide on systemic blood glucose and right ventricular remodeling in MCT-induced PAH rats. (A) Fasting blood glucose levels monitored during the 21-day administration period across the experimental groups (n = 4 per group). (B, C) Quantitative echocardiographic analysis of right ventricular free wall thickness (RVFW) (B) and right ventricular diastolic dimension (RVDd) (C) (n = 4 per group). (D) Representative M-mode echocardiograms of the right ventricle in each group. (E) Assessment of right ventricular hypertrophy evaluated by the Fulton Index (n = 4 per group). Data are presented as mean ± SD. *$P < 0.05$, **$P < 0.01$. (F) Representative Western blot images of basal protein expression levels of GLP-1 receptor (GLP1 R) and GIP receptor (GIP R) in total rat lung tissue, CON-PASMCs and MCT-PASMCs.

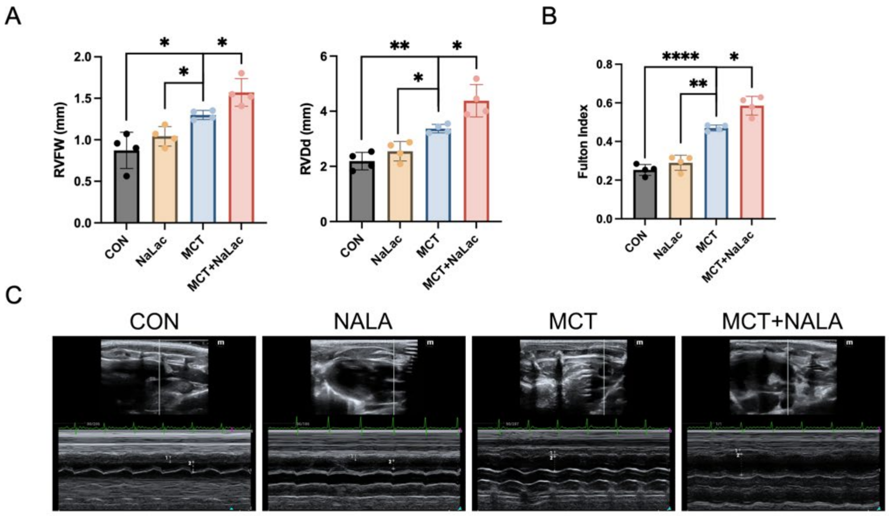

Supplementary Figure S2. The effects of exogenous lactate on right ventricular remodeling in MCT-induced PAH rats. (A) Quantitative echocardiographic analysis of right ventricular free wall thickness (RVFW) and right ventricular diastolic dimension (RVDd) across the experimental groups (*n* = 4 per group). (B) Assessment of right ventricular hypertrophy evaluated by the Fulton Index (*n* = 4 per group). (C) Representative M-mode echocardiograms of the right ventricle in each group. Data are presented as mean ± SD. *P < 0.05, **P < 0.01, ****P < 0.0001.

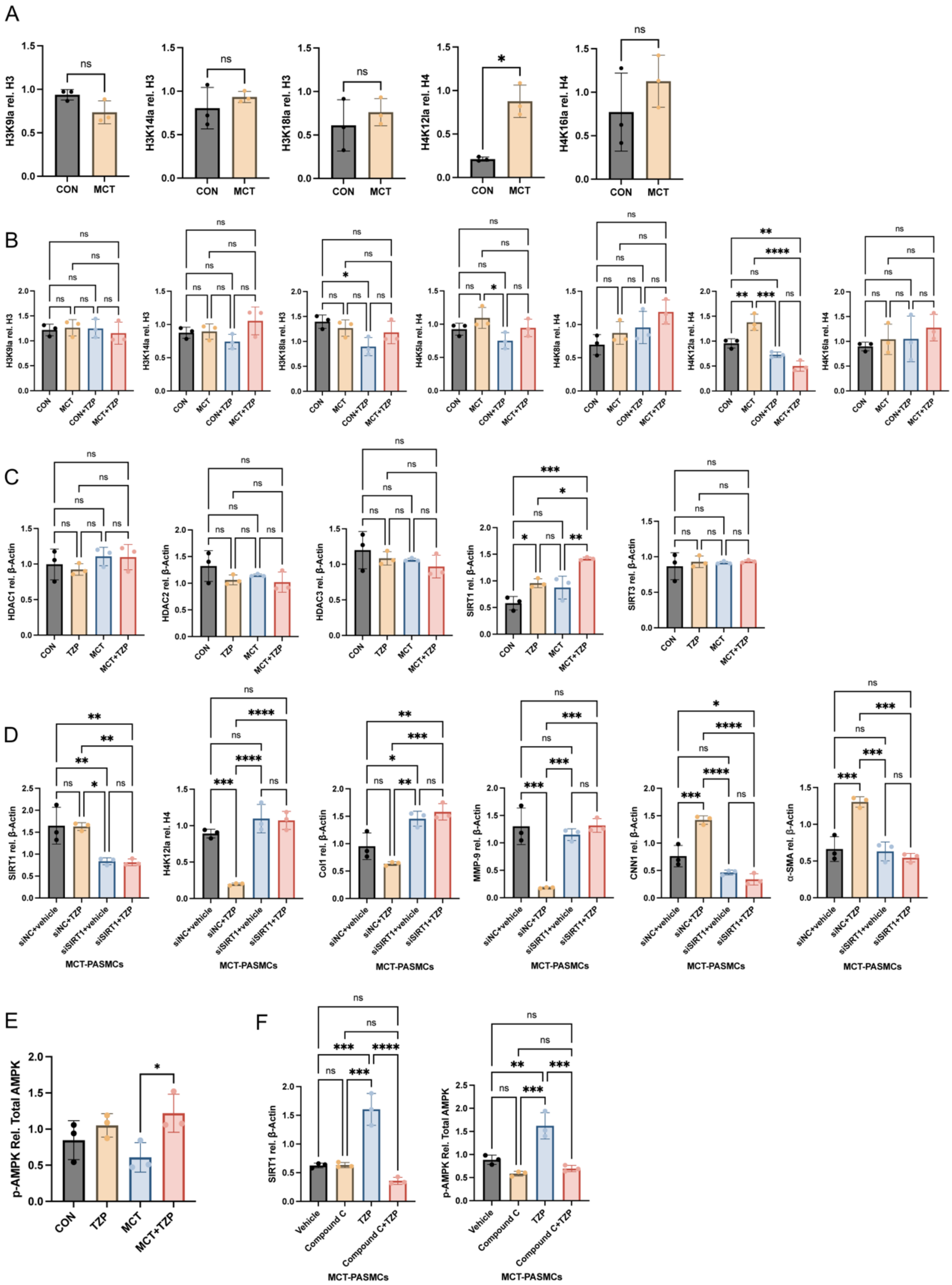

Supplementary Figure S3. Quantitative densitometric analysis of Figure 4B, C, F, G, J, K. (A) Densitometric quantification of specific histone H3 (H3K9la, H3K14la, and H3K18la) and H4 (H4K12la and H4K16la) lactylation marks relative to total H3 or H4 in CON or MCT rat pulmonary trunk tissues (n = 3 independent biological replicates). (B) Quantitative band density analysis of histone H3 (H3K9la, H3K14la, and H3K18la) and H4 (H4K5la, H4K8la, H4K12la, and H4K16la) lactylation levels relative to total H3 or H4 across CON, MCT, CON+TZP, and MCT+TZP groups of PASMCs (n = 3 independent biological replicates). (C) Densitometric quantification of HDAC1, HDAC2, HDAC3,

SIRT1, and SIRT3 relative to β-Actin across CON, TZP, MCT, and MCT+TZP groups (*n* = 3 independent biological replicates). (D) Quantitative analysis of SIRT1, Col1, MMP-9, CNN1, and α-SMA (relative to β-Actin), alongside H4K12la (relative to total H4), in MCT-PASMCs transfected with negative control siRNA (siNC) or SIRT1 siRNA (siSIRT1) with or without TZP treatment (*n* = 3 independent biological replicates). (E) Densitometric quantification of phosphorylated AMPK (p-AMPK) relative to total AMPK in CON, TZP, MCT, and MCT+TZP groups (*n* = 3 independent biological replicates). (F) Quantitative densitometry of SIRT1 (relative to β-Actin) and p-AMPK (relative to total AMPK) in MCT-PASMCs treated with the AMPK inhibitor Compound C in the presence or absence of TZP (*n* = 3 independent biological replicates). Data are presented as mean ± SD (*n* = 3 independent biological replicates). *P < 0.05, **P < 0.01, ***P < 0.001, ****P < 0.0001 indicate statistical significance between the connected groups; ns = not significant.

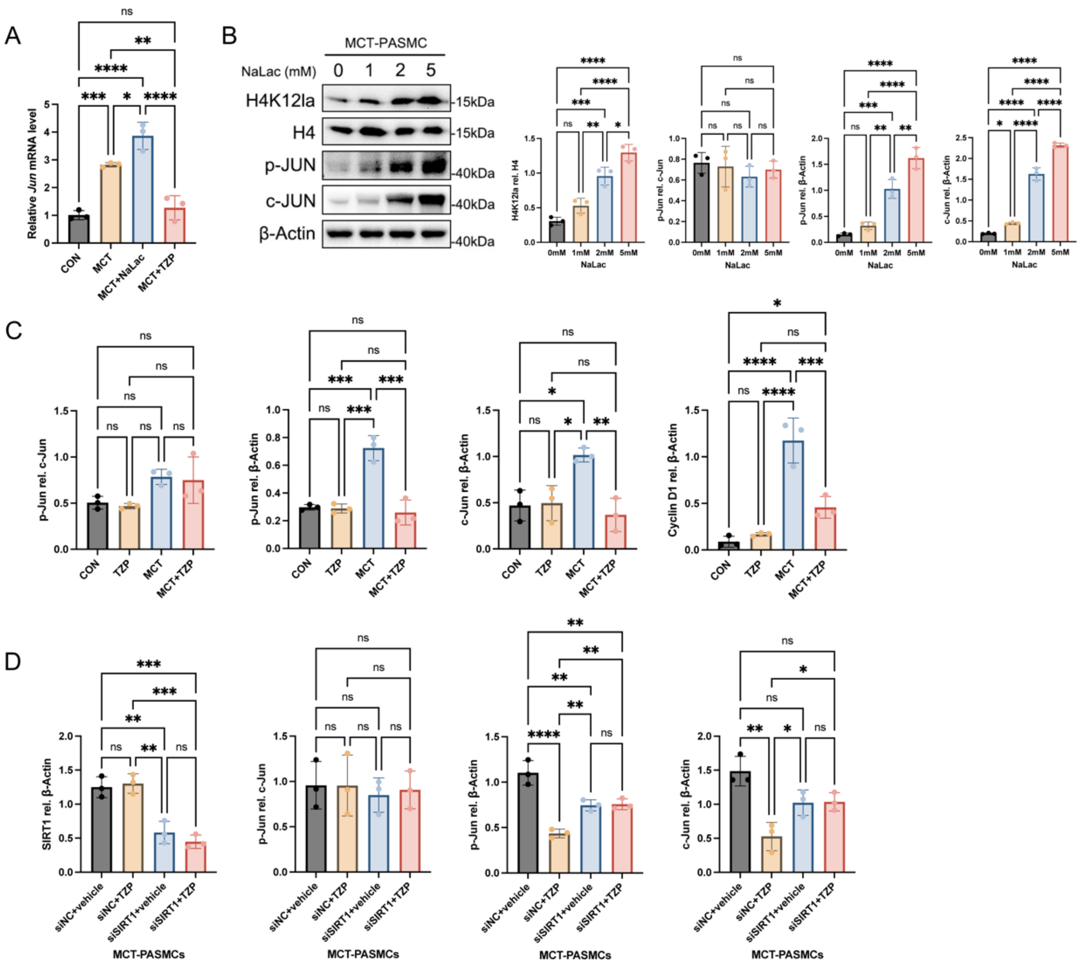

Supplementary Figure S4. Independent validation of lactate-driven *Jun* transcription, dose-dependent induction by NaLac, and quantitative densitometric analysis of Figure 5G, H. (A) Quantitative real-time PCR (qPCR) analysis of relative *Jun* mRNA expression levels in PASMCs across CON, MCT, MCT+NaLac, and MCT+TZP groups (*n* = 3 independent biological replicates). (B) Representative Western blot images and corresponding densitometric quantification of H4K12la (relative to total H4), phosphorylated c-Jun (p-Jun, relative to total c-Jun and β-Actin), and total c-Jun (relative to β-Actin) in MCT-PASMCs treated with indicated concentration gradients of NaLac (0, 1, 2, and 5 mM) (*n* = 3 independent biological replicates). (C) Densitometric quantification of p-Jun (relative to total c-Jun and β-Actin), total c-Jun (relative to β-Actin), and Cyclin D1 (relative to β-Actin) across CON, TZP, MCT, and MCT+TZP groups (*n* = 3 independent biological replicates). (D) Densitometric quantification of SIRT1, p-Jun (relative to total c-Jun and β-Actin), and total c-Jun (relative to β-Actin) in MCT-PASMCs transfected with negative control siRNA (siNC) or SIRT1 siRNA (siSIRT1) with or without TZP treatment (*n* = 3 independent biological replicates). Data are presented as mean ± SD (*n* = 3 independent biological replicates). *P < 0.05, **P < 0.01, ***P < 0.001, ****P < 0.0001 indicate statistical significance between the connected groups; ns = not significant.

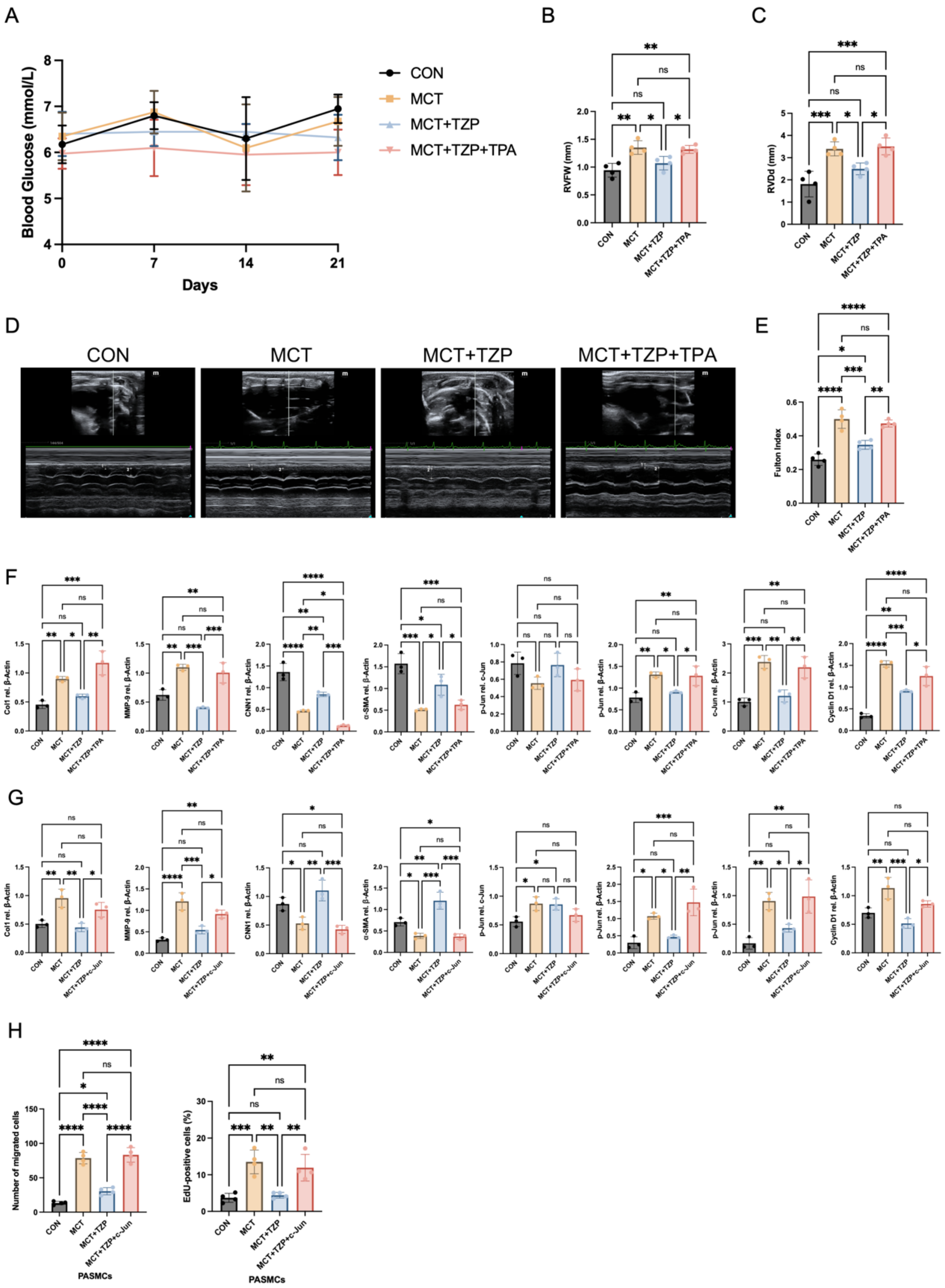

Supplementary Figure S5. Evaluation of systemic glycemic stability, right ventricular remodeling, and quantitative analyses of pharmacological and genetic c-Jun rescue experiments. (A) Fasting blood glucose levels monitored during the 21-day administration period across the experimental groups (n = 4 per group). (B, C) Quantitative echocardiographic measurements of right ventricular free wall thickness (RVFW) (B) and right ventricular end-diastolic dimension (RVDd) (C) (n = 4 per group). (D) Representative M-mode echocardiograms of the right ventricle in each group. (E) Assessment of right ventricular hypertrophy evaluated by the Fulton Index (n = 4 per group). (F) Densitometric

quantification of the Western blot bands from Figure 6J, displaying relative protein expression levels of Col1, MMP-9, CNN1, α-SMA, p-Jun (relative to total c-Jun and β-Actin), total c-Jun, and Cyclin D1 in MCT-PASMCs treated with TZP alone or combined with TPA (n = 3 independent biological replicates). (G) Densitometric quantification of the Western blot bands from Figure 6L, showing relative protein expression levels of Col1, MMP-9, CNN1, α-SMA, p-c-Jun (relative to total c-Jun and β-Actin), total c-Jun, and Cyclin D1 in MCT-PASMCs treated with TZP in the presence or absence of c-Jun overexpression plasmid (n = 3 independent biological replicates). (H) Quantitative analyses of migrated cells and EdU-positive cells (%) from Figure 6K in MCT-PASMCs treated with TZP with or without c-Jun overexpression (n = 4 independent biological replicates). Data are presented as mean ± SD. *P < 0.05, **P < 0.01, ***P < 0.001, ****P < 0.0001 indicate statistical significance between the connected groups; ns = not significant.
